# Modeling a Dynamic Printability Window on Polysaccharide Blend Inks for Extrusion Bioprinting

**DOI:** 10.1021/acsbiomaterials.2c01143

**Published:** 2023-02-27

**Authors:** Francesca Perin, Eugenia Spessot, Anna Famà, Alessio Bucciarelli, Emanuela Callone, Carlos Mota, Antonella Motta, Devid Maniglio

**Affiliations:** † Department of Industrial Engineering and BIOtech Research Center, University of Trento, Via Sommarive 9, 38123 Trento, Italy; ‡ European Institute of Excellence on Tissue Engineering and Regenerative Medicine Unit, Via delle Regole 101, 38123 Trento, Italy; § Department of Complex Tissue Regeneration, MERLN Institute for Technology-Inspired Regenerative Medicine, Maastricht University, Minderbroedersberg 4-6, 6211LK Maastricht, The Netherlands; ∥ Laboratorio RAMSES, IRCCS Istituto Ortopedico Rizzoli, via di Barbiano 1/10, 40136 Bologna, Italy; ⊥ ″Klaus Müller″ Magnetic Resonance Lab., Department of Industrial Engineering, University of Trento, Via Sommarive 9, 38123 Trento, Italy

**Keywords:** extrusion bioprinting, printability, hyaluronic acid, sodium alginate

## Abstract

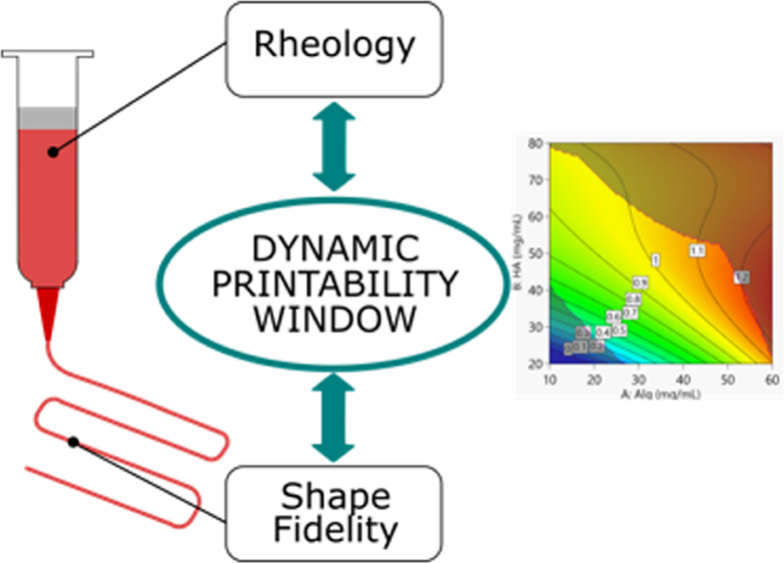

Extrusion-based bioprinting is one of the most widespread technologies due to its affordability, wide range of processable materials, and ease of use. However, the formulation of new inks for this technique is based on time-consuming trial-and-error processes to establish the optimal ink composition and printing parameters. Here, a dynamic printability window was modeled for the assessment of the printability of polysaccharide blend inks of alginate and hyaluronic acid with the intent to build a versatile predictive tool to speed up the testing procedures. The model considers both the rheological properties of the blends (viscosity, shear thinning behavior, and viscoelasticity) and their printability (in terms of extrudability and the ability of forming a well-defined filament and detailed geometries). By imposing some conditions on the model equations, it was possible to define empirical bands in which the printability is ensured. The predictive capability of the built model was successfully verified on an untested blend of alginate and hyaluronic acid chosen to simultaneously optimize the printability index and minimize the size of the deposited filament.

## 1 Introduction

Bioprinting is gaining an increased interest in the field of tissue engineering due to its potential in manufacturing scaffolds with precise geometric control compared to traditional biofabrication methods.^1^ Bioprinters exist with assorted setups, mostly adapted from existing additive manufacturing techniques (extrusion-based, ink-jet, or laser-assisted);^2^ nonetheless, the most widely diffused are the extrusion-based ones, thanks to their ease of implementation and affordability that brought several commercial bioprinters already on the market (e.g., BIO X, CELLINK; NovoGen MMX Bioprinter, Organovo; 3DDiscovery, RegenHU; BioAssemblyBot, Advanced Solutions).^1,3,4^ The major advantages of extrusion-based bioprinting (EBB) are the wide printing window in terms of viscosity (10^1^–10^13^ Pa·s^1^), high cell densities (>10^8^ cells/mL^5^), and remarkable achievable printing speeds.^6^ Yet, EBB is not free from drawbacks, such as the limited resolution (100–150 μm), the needle occlusion,^2^ and the requirements on gelation and solidification, reducing the choice of suitable materials.^6,7^ The desirable rheological behavior for a candidate EBB ink is shear thinning to favor extrusion at lower pressures, compensating for the high shear stress originated in the needle, and to ensure a rapid increase in viscosity after deposition to guarantee shape retention.^1,8^ Alternatively, several inks can be printed with high-quality shape fidelity by engineering the material to undergo fast gelation immediately after printing by external stimuli (i.e., temperature or pH changes, light irradiation).^9^ Advanced biomaterial inks are currently under development that can recapitulate physiochemical requirements for fabrication while promoting a cell-friendly environment, compromising between printability and biocompatibility.^10,11^ A univocal definition of the term printability has not been assessed yet, and for the purpose of this paper, the one suggested by Ribeiro et al.^12^ will be considered: “the possibility to extrude a hydrogel, and dispense it in a pattern with a satisfactory degree of shape fidelity, the latter indicating how the printed structure is matching the original CAD design”. This definition stresses the importance of a proper characterization of shape fidelity in evaluating printability while developing new inks. However, to date, there is no consensus on strategies to predict shape fidelity, and neither have standardized descriptions been recognized^12^ because the “New Test Method for Printability of Bioinks for Extrusion-based Bioprinting” from ASTM Committee WK72274 is still in draft form.^13^ This led to several published works tackling this issue by addressing ink properties (i.e., viscosity and other rheological properties^11,14^) or by focusing on the mere evaluation of the printing resolution and the shape retention of printed constructs on the basis of qualitative and/or quantitative evaluation of macro- and microscopic images.^10,12^ Despite the advancements in the printability assessment strategies, these approaches are often based on subjective parameters devised *ad hoc* for the system under study, are unable to provide a comprehensive analysis, and preclude the possibility of comparing different inks.^10,12^ In this context, the establishment of reproducible testing procedures would constitute a pivotal improvement for the assessment of ink printability.^15^ Steps in this direction have been taken by recent works providing tools to select printing parameters *a priori* from rheological analysis; however, much work is still needed to extend these tools to wider printing conditions and applications.^16,17^

In this work, a model ink was extensively characterized through rheological and shape fidelity empirical models aiming at creating a versatile tool for researchers working on engineering inks for extrusion bioprinting. The model ink was a blend of alginate and hyaluronic acid, polysaccharides commonly used for tissue engineering applications, suitable to be used for extrusion-based biofabrication techniques. The polysaccharide blend permitted one to investigate a wider spectrum of conditions with ease by varying the total concentration of the polymers and their relative amount. Eight conditions were selected to perform printability accuracy tests by carefully reviewing the literature, selecting quantitative methods only, and discarding the qualitative ones. Both the rheological and printability accuracy analysis results were collected into mathematical models, describing the ink behavior. In this context, a methodology that was widely used to tackle other additive manufacturing problems,^18,19^ namely, Design of Experiment (DoE), was applied. DoE is a statistical methodology that allows one to treat the printing process as a black box in which the printing parameters are considered as inputs (factors) and the property of the printed design as output (yields). Using this methodology, factors are varied following a well-defined strategy that allows one to maximize the information that can be extrapolated. In particular, DoE was used to build empirical equations relating factors and yields by imposing constraints on the equations of mass flow and on the spreading ratio. The printability window was defined as “dynamic”, as despite being built on the empirical model describing shape fidelity, it can be also applied to the rheological one, identifying the required rheology of inks to be printed under defined conditions. This results in a powerful tool to predict printing outcomes when manipulating polymers via functionalization or loading with additives (drugs, peptides, nanoparticles, and other components) that might affect the rheology and modify the printing outcome. This experimental work was focused on modeling printing parameters of monolayered structures with the assumption that once the dynamic printability window was built, it could be directly transferred to chemically modified polymers to build 3D constructs by photo-cross-linking each layer immediately after extrusion. The model not only identifies a range of parameters providing satisfying printing outcomes but also enables the optimization of the printing outcome via a desirability function, identifying the concentration and extrusion pressure necessary to achieve the optimal geometric accuracy. After validation of the models with an unmodified polymeric blend, the proposed tool was verified as able to predict the printing outcomes of a chemically modified version of the blend composed of methacrylated alginate and methacrylated hyaluronic acid. Moreover, the models allowed one to identify optimal printing parameters.

## 2 Materials and Methods

### 2.1 Ink Formulations

Alginic acid sodium salt from brown algae (BioReagent, suitable for immobilization of microorganisms) was purchased from Sigma-Aldrich (Darmstadt, Germany). Hyaluronic acid sodium salt (MW 200–400 kDa) was purchased from Glentham Life Sciences Ltd. (Corsham, UK). Sixteen different blend hydrogel compositions were formulated by mixing sodium alginate and hyaluronic acid powders dissolved in deionized water; the compositions of the inks are all reported in Table 1. Alginate and hyaluronic acid were methacrylated for printing of multilayer grids only following protocols from the literature.^20,21^ Solutions were thoroughly mixed and stored at 37 °C for 24 h. Before rheological characterization, all samples were centrifuged for 4 min at 25 °C and 3600 rpm to remove air bubbles. For printability tests, imaging of the hydrogels required the addition of 10% (v/v) food red dye *Mariarosa Rosso Color dolci* purchased from Rebecchi Fratelli Valtrebbia S.p.A (Piacenza, Italy). Staining of the hydrogels was necessary to easily threshold the images for batch analysis. Samples were subsequently transferred into 3 mL cartridges (CELLINK, Sweden) and centrifuged for 4 min at 25 °C and 3600 rpm prior to printing to remove bubbles.

**Table 1:** Polymer Solutions Tested for Rheology and Their Composition

	1ALG2HA	1ALG4HA	1ALG6HA	1ALG8HA
alginate, % (w/v)	1	1	1	1
hyaluronic acid, % (w/v)	2	4	6	8
	2ALG2HA	2ALG4HA	2ALG6HA	2ALG8HA
alginate, % (w/v)	2	2	2	2
hyaluronic acid, % (w/v)	2	4	6	8
	4ALG2HA	4ALG4HA	4ALG6HA	4ALG8HA
alginate, % (w/v)	4	4	4	4
hyaluronic acid, % (w/v)	2	4	6	8
	6ALG2HA	6ALG4HA	6ALG6HA	6ALG8HA
alginate, % (w/v)	6	6	6	6
hyaluronic acid, % (w/v)	2	4	6	8

### 2.2 Rheological Characterization

All the rheological evaluations were performed on a Discovery HR-2 hybrid rheometer (TA Instruments, New Castle, Delaware). The instrument was equipped with a stainless-steel cone/plate geometry (50 mm diameter, 2° cone angle, 100 μm truncation gap) and a Peltier plate for temperature control. The full list of the tested blends with their composition is shown in Table 1. All the inks were subjected to the following rheological measurements carried out in triplicates:

#### 2.2.1 Oscillatory Stress Sweep

The procedure was established based on a previous work^12^ by first preconditioning the samples with a shear flow peak hold (25 °C, 10 s, shear rate 300 s^–1^) to mimic the strong shear stress experienced by the ink during the extrusion process. Oscillatory stress sweep tests (shear stress ramp from 0.1 to 1000 Pa, 25 °C, 1 Hz) were conducted to investigate the viscoelastic behavior of the solutions by recording the storage (*G′*) and loss (*G″*) shear moduli behavior as a function of the applied shear stress. The results were analyzed with the TRIOS software (TA Instruments) to determine *G′* and *G″* in the linear viscoelastic region (LVE) and the yield stress (τ_y_) representing the onset point of the breakdown of the material microstructure and the end of the LVE region.^22,23^

#### 2.2.2 Rotational Shear Rate Sweep

The flow behavior properties of the inks were investigated in static mode. Samples were equilibrated at a temperature of 25 °C and exposed to rotational shear rate sweep from 0.1 to 300 s^–1^. Viscosity measurement curves were fitted with the Cross model (eq 1),^24^ in which η_0_ is the zero-shear viscosity (Pa·s), η_∞_ is the infinite rate viscosity (Pa·s), γ̇ is the shear rate (s^–1^), *k* is the characteristic polymer relaxation time (s), and *m* is a fluid specific parameter equal to (1 – *n*), where *n* is the shear thinning index of the power law model.
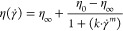
1

Additional tests were conducted to verify that the red dye used for enhancing imaging did not significantly impact the rheology of the inks (Figure S1).

### 2.3 Printability Characterization

This study was conducted using an extrusion-based bioprinter, BIO X (CELLINK, Boston, USA), equipped with standard pneumatic printheads. For all the printability evaluations, the ink solution was loaded into a 3 mL disposable cartridge (CELLINK) fitted with a polypropylene standard 25G conical nozzle. Printing was performed on a polystyrene Petri dish with a cartridge temperature of 30 °C, printing bed temperature of 25 °C, and deposition speed of 3.3 mm/s. The printing height was set as approximately two-thirds of the needle diameter and recalibrated before each print, as suggested in a previous work.^15^ Five different extrusion pressures were tested for each formulation, and each printing test is resumed in Table 2. All the G-codes used for the bioprinting trials and the Matlab procedures for bioprinted construct analysis are described in the following section and are available as Supporting Information.

**Table 2:** Ink Formulations and Relative Printing Pressures

label	alginate, % (w/v)	hyaluronic acid, % (w/v)	printing pressures (*p*_ext_), kPa
1ALG8HA	1	8	65, 70, 75, 80, 85
2ALG8HA	2	8	75, 85, 95, 105, 115
4ALG4HA	4	4	55, 60, 65, 70, 75
4ALG6HA	4	6	75, 85, 95, 105, 115
4ALG8HA	4	8	110, 120, 130, 140, 150
6ALG2HA	6	2	55, 60, 65, 70, 75
6ALG4HA	6	4	85, 90, 95, 100, 105
6ALG6HA	6	6	140, 145, 150, 155, 160

On the basis of a previous work,^25^ the first parameter evaluated in the printability assessment was the extrudability of the inks. Extrusion pressure (*p*_ext_) was slowly increased until the ink began to be extruded steadily to identify the minimum pressure necessary to extrude a uniform filament. The extrudability of the ink was then evaluated by weighting the amount of extruded ink in 40 s and by determining the gravimetric flow (Qm [mg/s]). This parameter was selected as a constraint in the following mathematical model because printing conditions resulting in null gravimetric flow would not have had a physical significance in modeling the other printing parameters.

The second parameter investigated was the uniformity ratio (UF), a factor described in another study^26^ to quantitatively determine the uniformity of extruded filaments, and defined by eq 2:
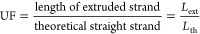
2

**Figure 1:**
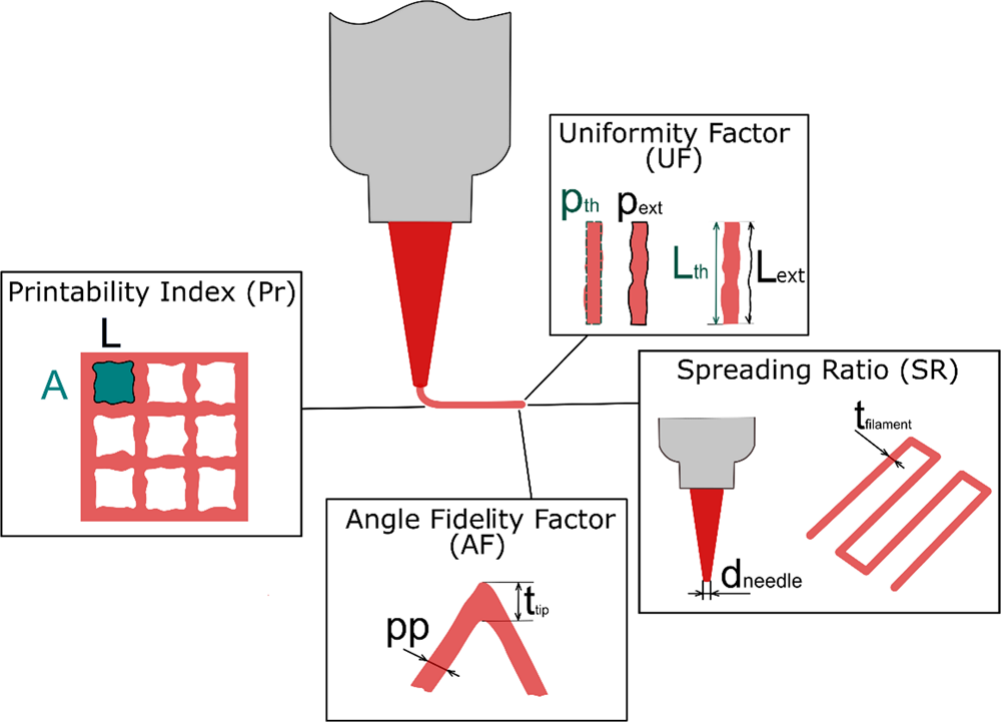
Schematic illustration of the definition of the printing parameters evaluated for the printability assessment: uniformity factor (UF), spreading ratio (SR), angle fidelity factor (AF), and printability index (Pr). The nature of all these parameters is thoroughly described in the Printability Characterization section of the Materials and Methods.

UF values close to 1 indicate smooth extrusion associated with hydrogels displaying a fluid-like behavior (*G*^″^ > *G*^′^), whereas higher values generally indicate nonuniform extrusion, typical of overgelated hydrogels. UF was measured by printing straight lines, acquiring images with a Dino-Lite AM7915MZTL digital microscope (Dino-Lite Europe, The Netherlands), and performing an automated image analysis with a Matlab procedure. To automate the analysis with more ease, UF definition was modified as described by eq 3. The parameter UF′ was computed by dividing the real perimeter of the filament in thresholded images (*p*_ext_) with a theoretical perimeter (*p*_th_), defined as a rectangle of the length *L*_th_ and width *t*_av_ (the average width of the filament). The difference between the two definitions of UF is schematically shown in Figure 1.
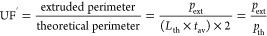
3

Then, the spreading ratio (SR) parameter was used to quantify the suitability of the inks to print structures with high precision and with limited coalescence and pore closure. SR was previously defined^27^ as “the width of the printed filament divided by the needle diameter” (see eq 4), and SR was computed on the same images acquired for UF using the same Matlab procedure.
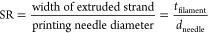
4

SR was used to limit the printability window by selecting a threshold value of 6. This was especially important at higher pressures and lower total polymer concentrations, at which SR would become excessively high, resulting in no accuracy when printing. Moreover, it has been reported that printing sharp angles often results in final geometries not complying with the desired one as a result of abrupt changing of the printing direction causing material accumulation at the tip of acute angles.^10,28,29^ This phenomenon makes SR inadequate as a parameter to evaluate the printing accuracy of complex geometries; therefore, an angle fidelity factor (AF) was introduced. AF was custom defined as the ratio between the thickness along the filament, pp, and the one at the tip of an angle, *t*_tip_ (eq 5), by printing 60, 90, and 120^○^ angles. A schematic illustration depicting the parameters involved in the angle fidelity factor evaluation is reported in Figure 1.
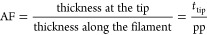
5

As a final control parameter, the printability index (Pr) was selected, which is often applied in printability studies.^30^ Pr quantifies the shape retention of an ink by investigating how much the square pores in a grid tend to round up after deposition; it was deduced from the definition of circularity of an enclosed area (*C*), as shown in eq 6 (where *L* represents the perimeter and *A* the enclosed area). Pr = 1 indicates perfectly square holes, Pr < 1 is a sign of more circular holes and merged filaments, and *Pr* > 1 indicates irregular shape and bumpy extrusion.
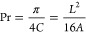
6

Pr was evaluated on 20 × 20 mm squares with 15% infill density by using a Matlab procedure and evaluating the 16 central squares of the grids.

To verify the possibility of applying the 2D printability assessment to build multilayered constructs by introducing a cross-linking step, multilayer grids of methacrylated ALG-HA blends were printed by photo-cross-linking each layer after deposition.

### 2.4 Segmentation and Image Analysis

**Figure 2:**
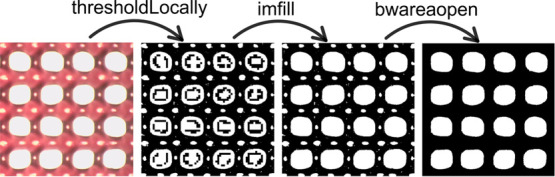
Schematic illustration of the image segmentation process and the Matlab functions used.

The analysis of the various images was carried out in batches with the Matlab software to speed up the procedure and guarantee a higher reproducibility than manual measurement by an operator. All the codes used for the analysis compute the various factors on segmented images. The segmentation procedure for the various kinds of constructs printed (lines, angles, and grids) was mainly based on the following functions, schematized in Figure 2:

*thresholdLocally(I, bs)* is a function available at MATLAB Central File Exchange, which performs Otsu thresholding on the image I with user specified blocksize (bs).^31^
*imfill(I,’holes’)* fills holes of a binary image I by identifying the background pixels that cannot be filled from the edges. This function was used only for manipulation of the grids images, as it was unnecessary for the other geometries.
*bwareaopen(I, P)* removes all the components of the binary image I that are smaller than P pixels. It is useful to remove small objects from binary images, such as the light reflections from the hydrogels producing small white spots in the binary images. Different limiting pixel sizes were selected for the various categories of constructs printed. All the codes, besides the one computing UF and SR, work by comparing different lengths of the images computed in pixels. However, for SR, measurements in microns are needed to be compared with the size of the needle; hence, before analysis, the pixel to micron aspect ratio was obtained through full image size calibration using ImageJ. All the pictures were cropped with ImageJ into suitable images needed for the written codes.

### 2.5 Statistical Methods

To extrapolate an empirical model, a Response Surface Method (RMS), previously used on different problems,^18,32−34^ has been adopted. The entire statistical analysis has been done by the use of the programming language R^35^ following the statistical strategy described in previous works.^18,19,32,34^ An initial analysis was done by calculating a correlation matrix in which the value of *r*^2^ was reported for couples of variables; this was used to verify the presence of linear correlation among them (correlation of the first order). Then, a model was built by selecting the significantly relevant factors tested by analysis of variance (ANOVA). The significant level was assigned as follows: *p* ≤ 0.1 (.), *p* ≤ 0.05 (*), *p* ≤ 0.01 (**), and *p* ≤ 0.001 (***). Only terms with *p* ≤ 0.1 were included in the model, whereas the model was considered significant with a *p* ≤ 0.05. The model function was chosen to achieve two scopes, to normalize the model residues and to make them patternless. To evaluate the goodness of fit of the model, the coefficient of determination (*r*^2^) was calculated. To model the rheological properties, the amounts in milligrams per milliliter of alginate (factor A) and hyaluronic acid (factor B) were modified as well as the printing pressure (factor C). The overall data set is reported in Table S1. The same factors were used to model the 2D printing yields (Pr, Qm, SR; data set in Table S2), and an additional factor, the printed angle (factor D), was added to quantify AF (data set in Table S3). A printability area was defined on the model by defining two conditions: the flow (Qm), defined as the material flow (in mg/s), was imposed to be higher than 0, and the spreading ratio (SR), defined as the ratio between the filament diameter and the nozzle diameter, was lower than 6. Based on the models, a desirability approach has been used to predict the “best condition” in which Pr is equal to 1 and SR is minimized. The optimization has been done by a numerical method based on desirability functions. These functions are in the [1,0] range, where 1 represents the optimum solution. One of these functions was assigned to each of the considered yields. The following notation have been used: *Y*_i_ the specific yield, *d*_i_ the corresponding desirability function, *U*_i_ and *L*_i_ the maximum and the minimum value of the yield, respectively. For the minimization of *Y*_i_, the function is reported in eq 7 (used in the case of SR); to set the yield on target, the function is reported in eq 8 (used in the case of Pr). The overall desirability was then calculated as the geometric mean of these functions and reported in eq 9 with *k* equal to the total number of yields (in our case, 2). *D* was plotted against the process factors to find its maximum value corresponding to the best solution.
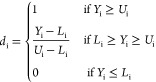
7
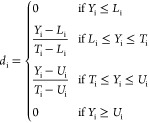
8
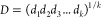
9

## 3 Results

### 3.1 Rheological Characterization

**Figure 3:**
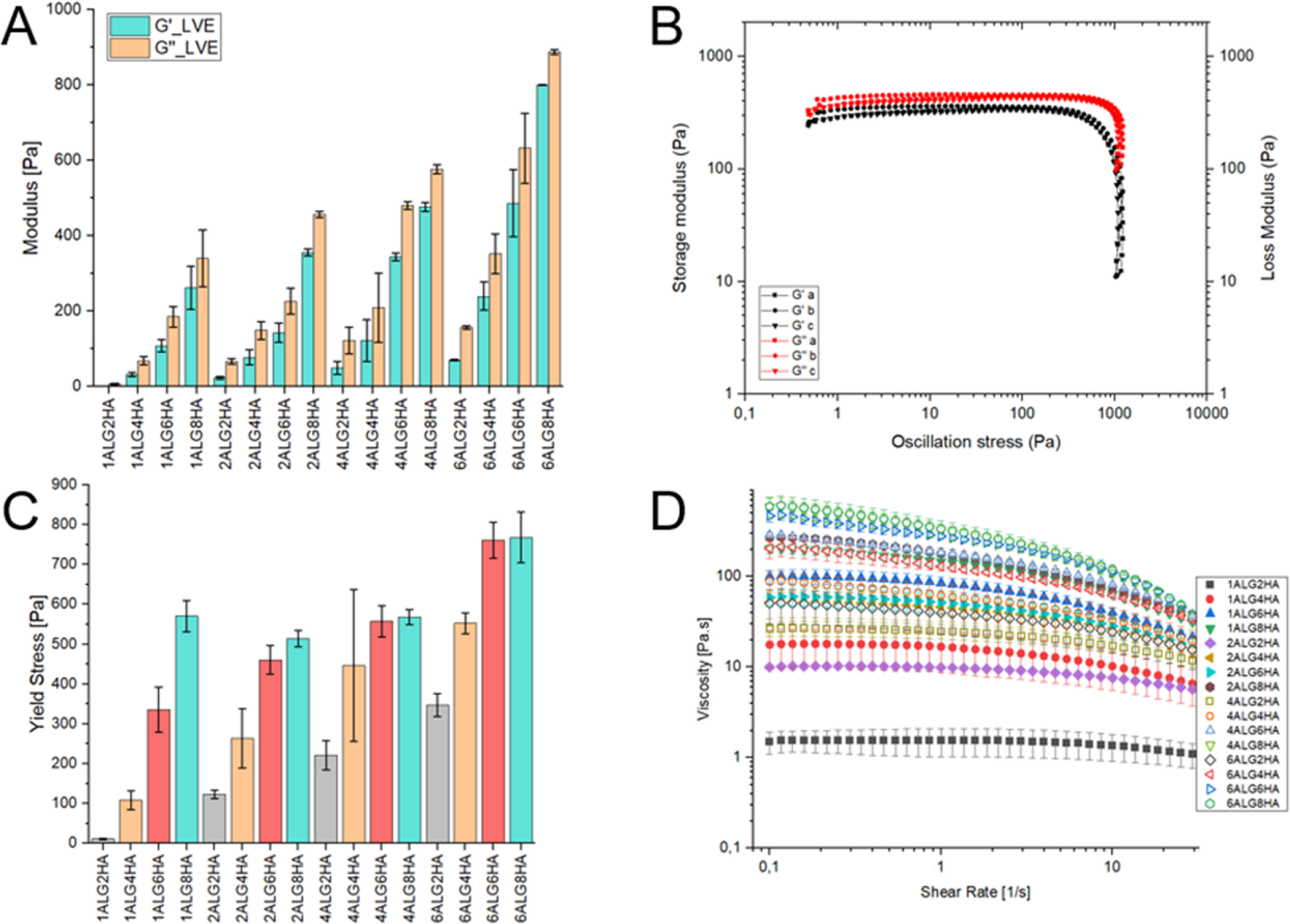
(A) Mean LVE region moduli acquired from oscillatory stress sweep tests for all tested conditions; (B) oscillatory stress sweep tests for 2ALG8HA, as representative curves for all the results; (C) average yield stress acquired from oscillatory stress sweep; and (D) rotational shear rate sweep average curves for all the tested conditions.

All the investigated samples behaved as viscous fluids, with the loss modulus (*G″*) being greater than the storage one (*G′*), as shown in the oscillatory stress sweep tests, results of which are shown in Figure 3A,B. The dominance of the loss modulus was expected because the viscous behavior of both polysaccharide aqueous solutions is well documented in the literature.^36−38^ There was a noticeable correlation between the total amount of polymer in the solution and the loss and storage plateau moduli in the LVE, as well as the onset of yielding, as shown by τ_y_ in Figure 3C. The total polymer concentration affected the values of viscoelastic moduli, which increased when moving from 3% (w/v) to 14% (w/v) total concentration. The oscillatory stress sweep curves for the three replicates of 2ALG8HA are reported in Figure 3B as representatives because all the curves were similar (the other curves are shown in the Figure S2). The values for *G′* and *G″* reported correspond to the values in the plateau of the linear viscoelastic region (LVE), whereas yielding was detected using the *Onset point* function in the TRIOS software (which traces tangents of the curve before and after yielding and identifies the onset of the phenomenon in their intersection). Moreover, the rotational shear rate sweep test evidenced a shear thinning behavior for most of the blends, with the viscosity decreasing when increasing the shear rate (Figure 3D). A higher concentration of polymer in the solution enhanced the shear thinning behavior. The viscosity curves have been fitted by using the Cross model (eq 1), obtaining the zero-rate viscosity (η_0_), the consistency index (*k*), the Cross index *m* (*m =* 1 – *n*), and the infinite-rate viscosity (η_∞_). η_∞_ was found to be neglectable for all the investigated conditions. All the tests presented a sharper decrease of the viscosity coupled with decreasing shear stress when approaching 100 s^–1^. This trend has been reported to be representative of edge fracture;^39^ hence, the final part of the curve was disregarded.

### 3.2 Printability Characterization

Out of the 16 conditions studied in the rheological model, only 8 were selected for printing. The conditions with the lowest total polymer content were disregarded, as they did not extrude in the form of filaments, but as droplets, and were hence deemed not suitable for printing. On the other hand, inks with a total polymer concentration above 12% w/v were disregarded because of the excessively high pressures needed to extrude them and the general difficulties in handling (difficulties in dissolution, hard to uniformly mix the ink, almost impossible to remove bubbles from the cartridges even with thorough centrifugation). The eight selected inks could successfully be extruded as continuous filaments at pressures below 200 kPa, being processable with the BIO X bioprinter. Measurements on the mass flow rate (Qm) showed a clear increasing trend with the extruding pressures (Figure S3A), with less concentrated inks extrudable at lower pressures and with higher mass flow rates. Moreover, most of the tested compositions displayed a uniformity ratio ranging from 1.0 to 1.1, indicating uniform extrusion of straight lines without bumps. This was expected because all the printed formulations were not pre-cross-linked and presented ideal filament formation during extrusion. The uniformity factor was generally unaffected by increasing the printing pressures or the total polymer content (Figure S3B**)**.

To optimize the printing quality, the spreading ratio (SR) was evaluated. The calculated SR suggested that all the tested inks consistently deviate from the intended width, ideally identified with the diameter of the printing nozzle. In fact, the calculated SR was higher than 1 for all the mixtures (range, 3.0–7.8). Nevertheless, this issue is often reported in the literature, with the minimum value for spreading ratio rarely below 2.^27,40,41^ The high spreading ratio values are a consequence of the postprinting relaxation of polymer chains, which move from a well-oriented state during extrusion to randomly oriented after processing. Moreover, SR showed an increasing trend with higher extrusion pressures, similarly to Qm (Figure S3C). Thus, the selection of the lowest pressure is desirable for the fabrication of printed structures with higher precision.

The other important parameter for the optimization of the printing resolution is the evaluation of material accumulation at the tip of sharp angles by means of the angle fidelity factor (AF). As expected, AF was found to be much higher when printing sharp angles (60 and 90°), whereas when printing 120° angles, it was close to 1 (see Figure S4). This clearly indicates the overlapping of the polymer strand during printing and suggests punctual G code editing to increase their printing accuracy (i.e., speeding up the printing velocity on the tip of the angles to limit the phenomenon). Because the accumulation of the material at the tip is caused by overlapping of material during the head movement, AF presents a similar behavior as SR.

**Figure 4:**
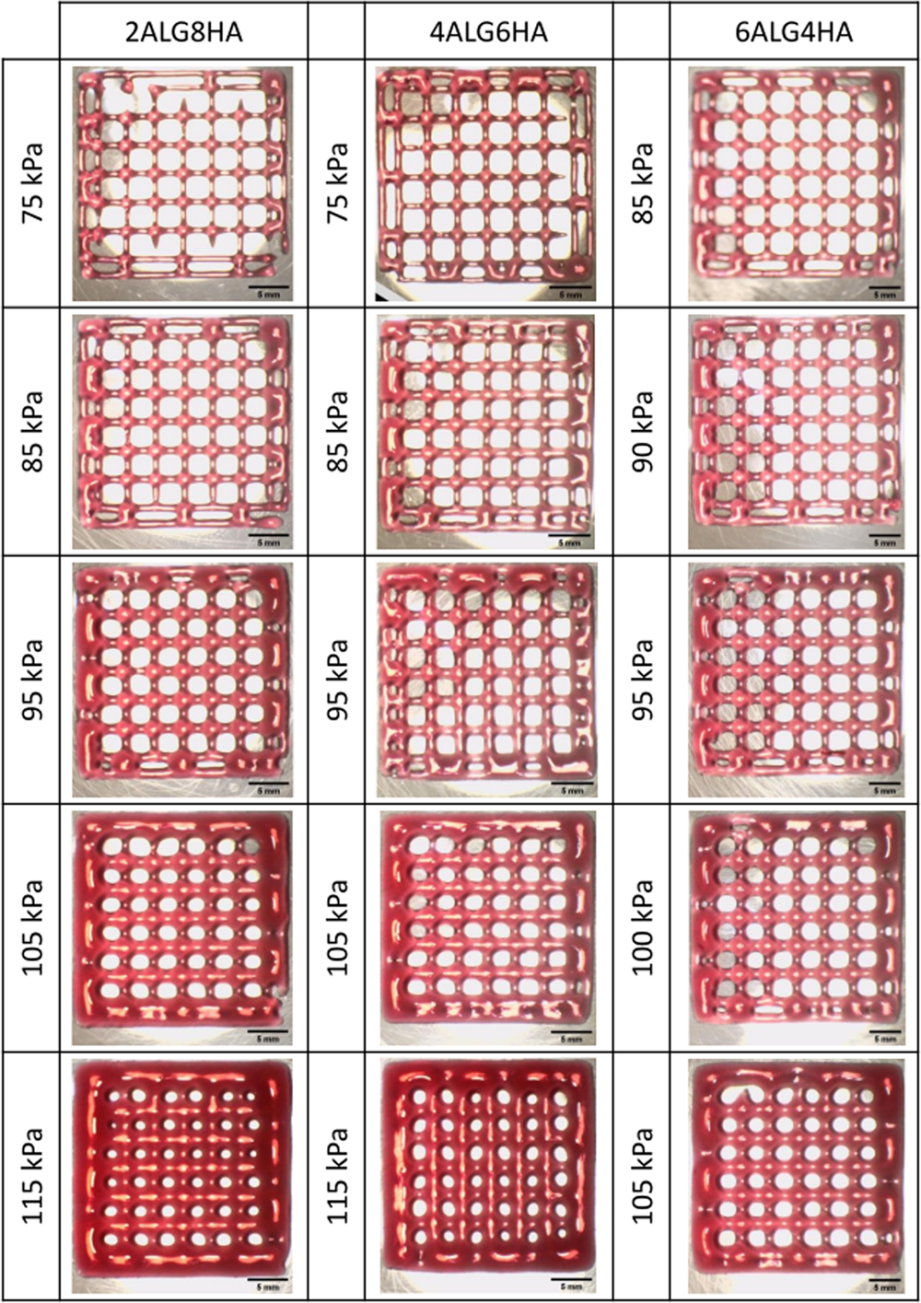
Example of the effect of the increasing extrusion pressure on grids of three different compositions.

The shape retention of the inks was quantified by the printability index (Pr). The obtained Pr values were lower than 1 for all the blends, hence suggesting an undergelation status. As confirmed by rheological tests, all the samples were characterized by a predominantly viscous behavior, resulting in time-limited mechanical stability without crosslinking. Nevertheless, most of the printability indices for all the formulations lie in the 0.9–1.2 range, suggesting acceptable printing performances for all the self-standing inks (see Figure S3D). Increasing the printing pressure caused the grid holes’ shape to deviate more and more from a square to a round one (see Figure 4).

The progressive increase of pressure causes more material to be deposited and an increase in the SR, consequently lowering the accuracy of the printed grids and causing angles to round up.

With the aim to verify the possibility of printing multilayered structures, we switched to a photo-cross-linkable blend of alginate-methacrylated (AL-MA, degree of functionalization 1.1%) and hyaluronic acid-methacrylated (HA-MA, degree of functionalization 29.6%). NMR analysis of the methacrylate polysaccharides is available in the Supporting Information (Figure S9 and Table S4). Rheological analysis showed that the methacrylation process did not significantly affect the rheology of the polysaccharides (Figure S10); hence, the methacrylated blends were processed using the same printing parameters as the nonmethacrylated counterparts.

**Figure 5:**
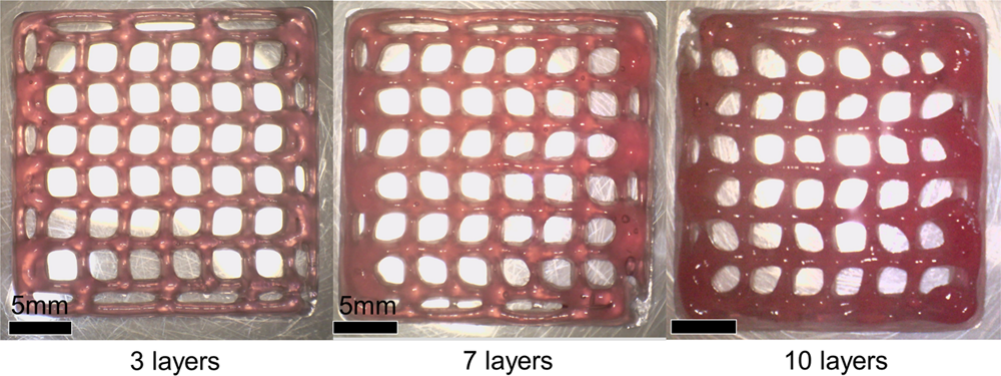
Multilayered grids of 6AL-MA6HA-MA printed at 145 kPa.

The multilayered grids could be printed with satisfying quality, as shown in Figure 5, even if the introduction of a UV exposure step after the deposition of each layer was necessary to stabilize the shape of the 3D constructs. Additional photos of the multilayered constructs and information on the printing and photo-cross-linking process are available in Figure S11 and Table S5.

### 3.3 Statistical Model

**Figure 6:**
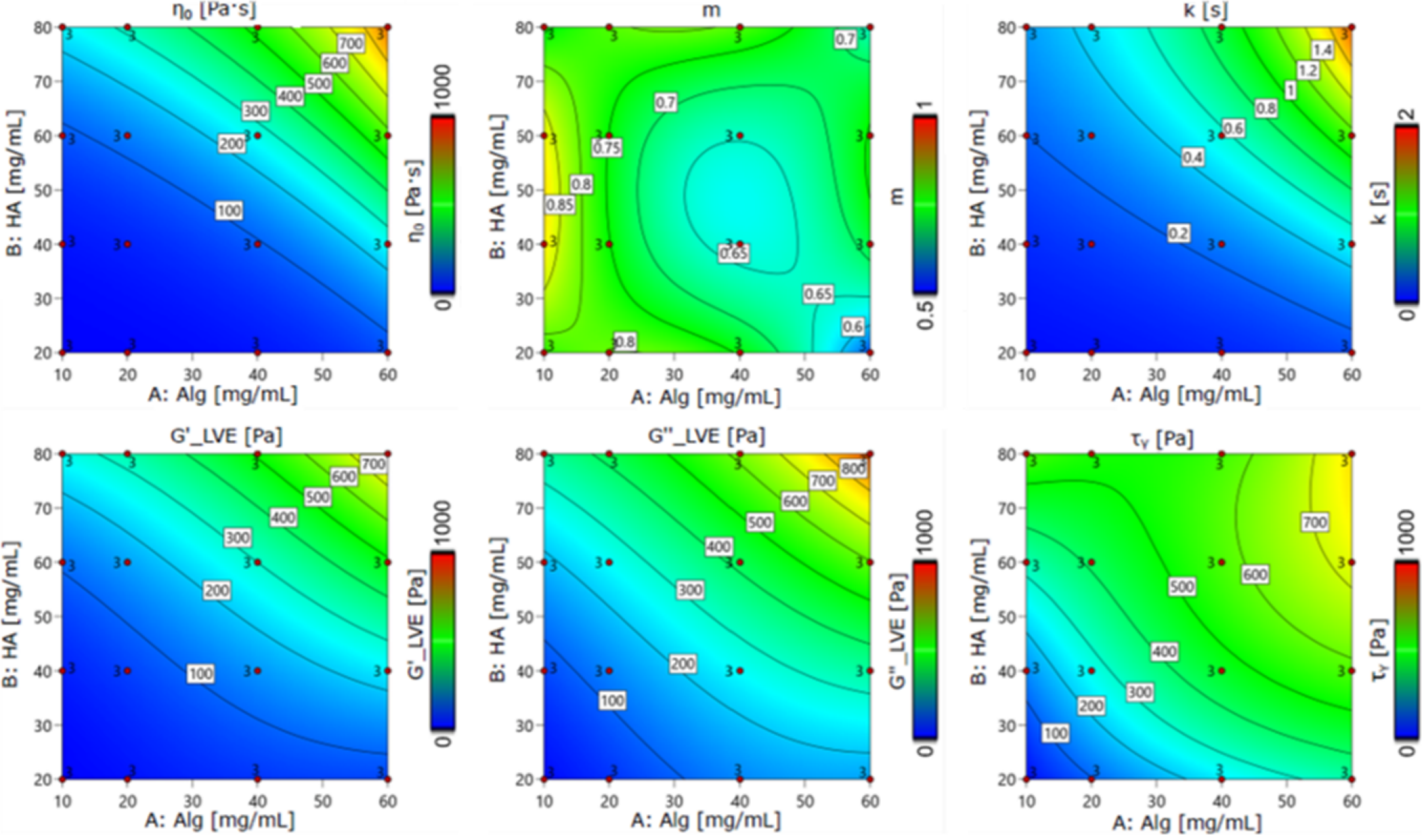
Contour plots representing the mathematical model built on the rheological analysis.

The empirical model built on the rheological data is schematized in the contour plots in Figure 6, where η_0_, *m*, and *k* stand, respectively, for the zero-rate viscosity, the flow index, and the relaxation time from the Cross-law fitting of the rheological measurements on viscosity. Below, G*′*_LVE and G*″*_LVE stand for the storage and loss modulus, respectively, in the linear viscoelastic region (LVE) of the stress oscillation curves, whereas τ_y_ represents the oscillation stress at the onset of yielding (representing the end of the viscoelastic region). The results of the model are in good accordance with those expected from the rheological analysis. The viscosity curves fitted with the Cross model displayed both a high viscosity and a shear-thinning behavior. The fitting parameters η_0_, *k*, and *m* were used for the model construction and compared with the variation of the polymers in the blend, suggesting high correlation between η_0_ and *k* with the relative hyaluronic acid/alginate concentration (as reported in Figure S5). However, the index *m* did not show a marked correlation with the ratio of polymer in the blend or the total concentration of polymer. Additionally, all the tested blends displayed a viscous-like behavior, having *G″* > *G′*, and were highly correlated in the model with the blend composition as for the yield stress (as reported in Figure S5).

**Figure 7:**
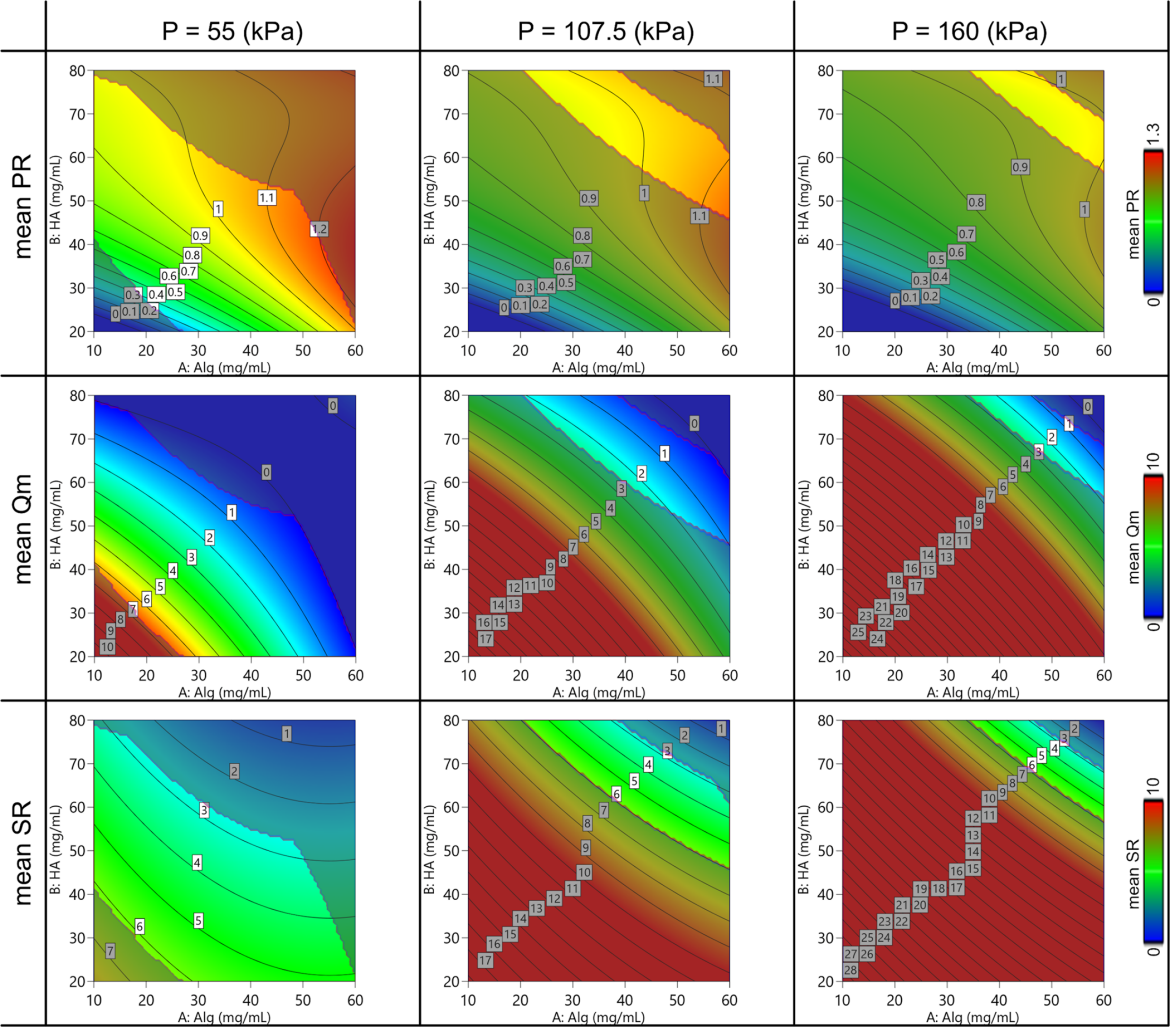
Contour plot of the model predicting the mean value of 2D printing parameters (Pr, Qm, and SR) varying the Alg-HA amount and the printing pressure.

**Figure 8:**
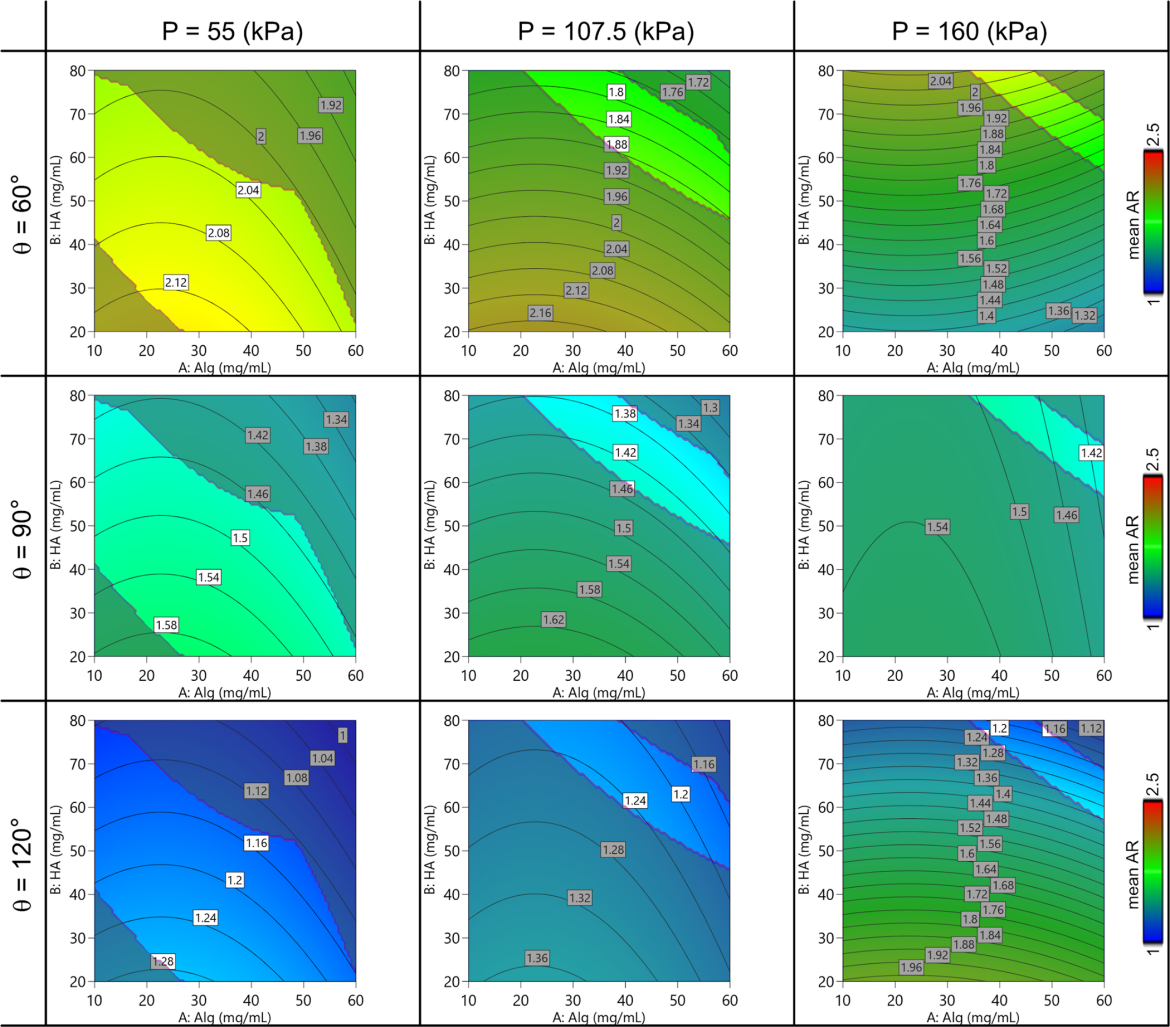
Contour plot of the model predicting the mean value of AF varying the Alg-HA amount, the printing pressure, and the printed angle.

Regarding the printability assessment study, the empirical model is schematized in the contour plots in Figures 7 and 8. A general behavior was observed for all the parameters depending on the printing pressure and the polymer concentration. The dynamic printability window determined by imposing constraints on the mass flow rate (Qm > 0) and on the spreading ratio (SR < 6) is highlighted.

**Figure 9:**
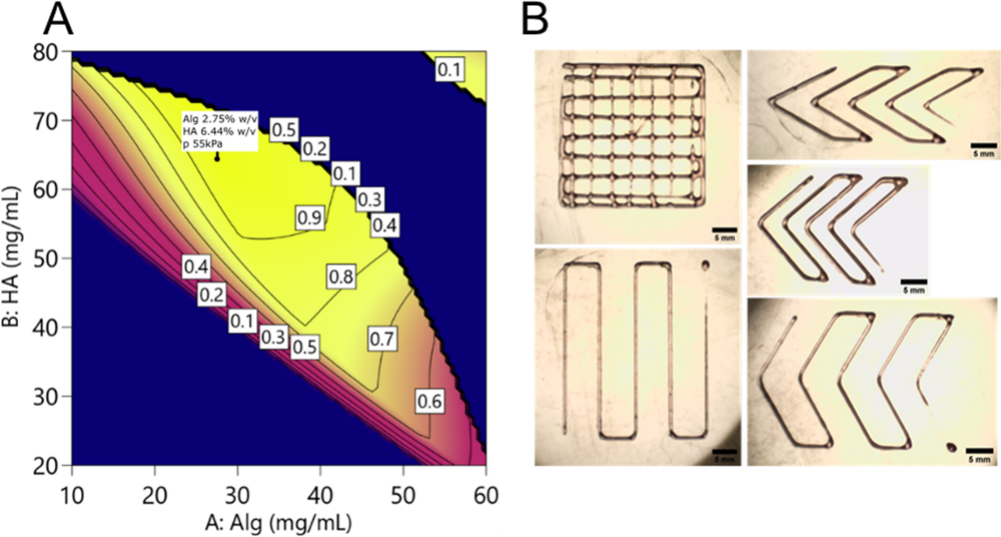
(A) Desirability function: the predicted best point is shown in the flag (Alg 2.75%, HA 6.4%); the best predicted pressure was 55 kPa. In these specified conditions, the desirability function is equal to 1. (B) Grid, lines, and angles printed under the optimal conditions found through the desirability function.

The validity of the model as a guideline for printing was tested on a critical point defined as a point in which the predicted Pr was equal to 1 and the value of SR was minimized. By using the desirability approach, this point was individuated (alginate 2.7% w/v, hyaluronic acid 6.6% w/v, printing pressure 55 kPa; Figure 9A) and tested (Figure 9B). The results of this tests are reported in Table 3. All the collected values were inside the 95% confidence band, demonstrating the validity of the model in predicting the printing outcome and the parameters related to the gel rheology.

**Table 3:** Predicted Values of the Printability and Rheological Models (Predict) and the Correspondent Values Obtained by Trial (Data Mean)^a^

analysis	predict	std dev	*n*	95% CI low	data mean	95% CI high
Pr mean	1.000	0.063	1	0.769	1.029	1.231
Qm mean	0.466	0.509	1	–0.609	0.120	1.541
SR mean	2.679	0.611	1	1.025	3.959	4.334
η_0_‡	257.556	105.716	1	104.497	215.805	543.277
*m*	0.715	0.093	1	0.51845	0.666284	0.91286
*k*‡	0.429	0.260	1	0.116178	0.715256	1.16021
G′_LVE‡	250.793	39.445	1	173.154	249.26	339.137
G″_LVE‡	356.975	55.790	1	247.638	321.31	481.15
τ_y_	491.346	60.2449	1	363.653	500.26	619.039

a Being that the data means are inside the 95% band, all the models resulted to be predictive.

## 4 Discussion

Biomaterial inks need to fulfill different requirements, such as extrudability and shape retention after extrusion, to be processed with a bioprinter. Given the absence of guidelines on how to define such properties *a priori,* currently, the optimization of ink formulation for extrusion bioprinting is a time-consuming procedure based on trial-and error methods to define the optimal printing parameters for each ink.

This work focused on overcoming this time-consuming procedure by proposing a set of empirical mathematical models, highlighting a dynamic printability window as a function of the printing pressure and the composition of a blend ink for EBB. Two sets of mathematical models were built: one based on an extensive rheological analysis and the other based on common quantitative parameters to evaluate the shape fidelity of planar constructs.^29^ Rheological properties are the key physicochemical features to comprehend the printability of a hydrogel.^26^ During the extrusion bioprinting process, the ink can be regarded as a fluid, passing from a resting state in the cartridge to a flowing state under a high shear stress in the nozzle during extrusion and ideally recovering the original rest state after extrusion. The properties of the ink during all these steps can be quantitatively described with viscosity, viscoelastic shear moduli, elastic recovery, and yield stress.

When designing inks, usually, a shear thinning behavior combined with high viscosity at low shear stresses is preferred. A shear thinning behavior results in an easier extrusion induced by the drop in viscosity in the nozzle caused by the high shear stress and thus the consequent alignment of the polymer chains. At the same time, this behavior implies a quick recovery of the viscosity after deposition (no shear stress), which aids in preserving the geometric accuracy.^42,43^ The viscosity of a hydrogel can be manipulated by changing the concentration, the molecular weight, and the conformation of the polymer (e.g., linear to branched). Besides the viscosity, the viscoelastic behavior of biomaterial inks should be considered for a complete analysis of the material. In fact, the storage (*G′*) and loss moduli (*G″*) characterize the material elastic shape retention and the viscous flow, respectively. Usually, *G′* is considered as the main parameter influencing the printability; however, recent studies evidenced that *G″* has a role in the behavior of the ink during printing.^26^ Additionally, another relevant parameter for the ink printability estimation is the yield stress, which can be defined as the minimum stress required for an ink to flow.^44^ Hence, a belated yield point ensures a higher filament quality^42^ because the deposited material with a relatively high yield stress will not spread quickly as a result of the low forces applied. All the presented parameters could aid in the optimization of ink printability and resolution and have been used in the DoE presented in this work. For the construction of a mathematical model, different tests to describe the properties of the ink have been selected, such as an amplitude stress test to quantify the storage modulus and the loss modulus, a yield stress of the ink, and a strain sweep test carried out to assess the shear thinning property of the material. Despite being a great tool to evaluate the extrudability of a material, rheological properties are not sufficient to define printability, as they are unable to correctly predict the geometric accuracy of the constructs. In this experimental work, five parameters were selected after careful literature review to quantify the extrudability of the studied inks and the resulting shape fidelity of single-layered constructs. As a starting point, the extrudability and filament formation of the inks were evaluated following the steps described in the literature^25^ to identify the minimum pressure necessary to extrude a continuous filament and to measure the mass flow rate at different pressures. Once filament formation was assessed, shape fidelity was evaluated on different printed geometries, such as straight lines to evaluate uniformity of the filament, grids to evaluate the self-standing ability of the inks, and finally angles to assess accumulation factors when printing complex geometries. Given the fluid-like behavior of the inks assessed during rheology and the good filament formation assessed previously, nonuniform extrusion was expected for the inks. This was confirmed as UF was close to 1 for all the inks at all the investigated pressures. Printed lines were used to also evaluate the spreading ratio, which instead resulted to be quite high. Ideally, an ink would be extruded with exactly the same size as the nozzle to achieve maximum geometric accuracy. This is not the case for hydrogels, with SR rarely being below 2 as reported in the literature.^27,40,41^ The obtained SR was in a wide range from 2.5 to 7.5 (see Figure 7), and these values evidenced a strong dependence of this parameter to the extrusion pressure. This is a not surprising result because, in accordance with their rheological properties, inks are easily extruded but tend to collapse onto the printing surface after deposition. The trend of SR with the pressure is in good accordance with the behavior observed through the mass flow rate measurements. Similar results were evidenced from the measurement of Pr after printing grids. Normally, it would be desirable to print the ink at the lowest pressure possible, especially in view of a possible inclusion of cells, to prevent excessive stress damage. It was decided to test five different printing pressures to build a wider mathematical model and to have a clear idea of their effect on the printing accuracy. As mentioned in the Materials and Method section, the mathematical model built on the collected data on printability was the base to build a dynamic printability window as a function of the printing pressure. To build the printability window, we imposed limits on the mass flow rate (Qm > 0 mg/s) to exclude nonextruding conditions and on the spreading ratio (SR < 6) to avoid taking into consideration printing conditions causing excessive filament collapse onto the surface. If deemed necessary, the printability window could be modified by imposing more restrictive conditions (i.e., thinner filaments by reducing SR).

The dynamic printability window was verified as successful to predict the printing outcomes of untested concentrations of the blend and to optimize printing outcomes as shown in Figure 9B. This implies the possibility of avoiding future trial-and-error tests to assess the printability of alginate and hyaluronic acid blends. Moreover, once the velocity, needle size, and temperature are defined, the printability window can be also applied to the rheological models, allowing one to identify a range of parameters that correspond to printable inks at a given extrusion pressure, as shown in Figures S6–S8. This makes the proposed tool extremely adaptable to variations of the tested inks, such as modified alginate and hyaluronic acid. In fact, both polysaccharides are reported in the literature with various modifications to introduce either covalent cross-linking routes (i.e., click-chemistry,^45^ photo-induced,^46,47^ and enzymatic^48,49^) or biological cues (i.e. peptides^50^). The dynamic printability window allows one to quickly determine the printing conditions of such inks via rapid rheological measurements so that the polymer modifications can be studied and optimized for their target applications, avoiding long trial-and-error printing optimization procedures. As a proof of concept, this was verified by printing the AL-MA/HA-MA blend, which was photo-cross-linked after the deposition of each layer to build 3D constructs.

## 5 Conclusions

The mathematical model presented in this work was able to predict the printability of a hyaluronic acid/alginate blend as a function of the extrusion pressure with set printing parameters (bioprinter, nozzle, and printing speed). The capability of the dynamic printability window in predicting the printability of untested alginate–hyaluronic acid blends and the possibility of using a desirability function to find the optimal printing parameters were verified. The versatility of this model allows its use as a tool for engineering new inks for EBB, avoiding the time-consuming traditional optimization process. This tool could be especially useful for researchers working on the development and engineering of novel inks for EBB. The use of an empirical model over a physical one allows one to apply the found printability window under any circumstances in which a change in the rheology is present without remodeling the whole system. For instance, the introduction of additives or temperature or pH changes can be addressed by repeating the rheological tests in the modified conditions and using the measured parameters as input to find the printing pressure and predict the shape fidelity of the constructs. It must be noted that this is not valid for excessive variations of the rheological behavior, such as deviations from the Cross model or disappearance of the yield stress.

There is room for improvement of this model because the only printing parameter considered in this study was the extrusion pressure even though there are numerous others affecting the outcome of the process, such as the size and shape of the needle, the temperature, and the velocity. This work is a starting point for building a much more sophisticated model including additional printing parameters. This will inevitably increase the complexity because some parameters will have a synergistic effect on the printing quality parameters. As an example, the printing velocity can be a precious tool to limit the spreading ratio. Alternatively, the obtained model could be used as a canvas to optimize some specific printing parameters.

## Supporting Information Available

Comparison of viscosity measurements (Figure S1); storage and loss moduli (Figure S2); average mass flow rate, uniformity ratio, spreading ratio, and printability index (Figure S3); angle fidelity factor (Figure S4); correlation matrix of the data set (Figure S5); data set used to fit the empirical model of the rheological properties (Table S1), the empirical model of the 2D printing yields (Table S2), and the model of the angular 2D printing yields (Table S3); contour plots of the model (Figures S6–S8); ^13^C CPMAS NMR spectra (Figure S9); methacrylation degree (Table S4); viscosity measurement (Figure S10); pictures of some multilayered methacrylated blend grids (Figure S11); and parameters for the photo-cross-linking of the methacrylated multilayered constructs (Table S5) (PDF)
