# Supplementary material for: Modeling a Dynamic Printability Window on Polysaccharide Blend Inks for Extrusion Bioprinting
Source: ACS Biomater Sci Eng. 2023 Feb 27;9(3):1320–31. doi: 10.1021/acsbiomaterials.2c01143 (PMC10015426; doi:10.1021/acsbiomaterials.2c01143)
Supplement: Supplementary file 1 — ab2c01143_si_002.pdf [file ab2c01143_si_002.pdf]

Supplementary Information: Modelling a dynamic printability window on polysaccharides blend inks for extrusion bioprinting

Francesca Perin and Eugenia Spessot, Anna Famà, Alessio Bucciarelli, Emanuela Callone, Carlos Mota, Antonella Motta and Devid Maniglio

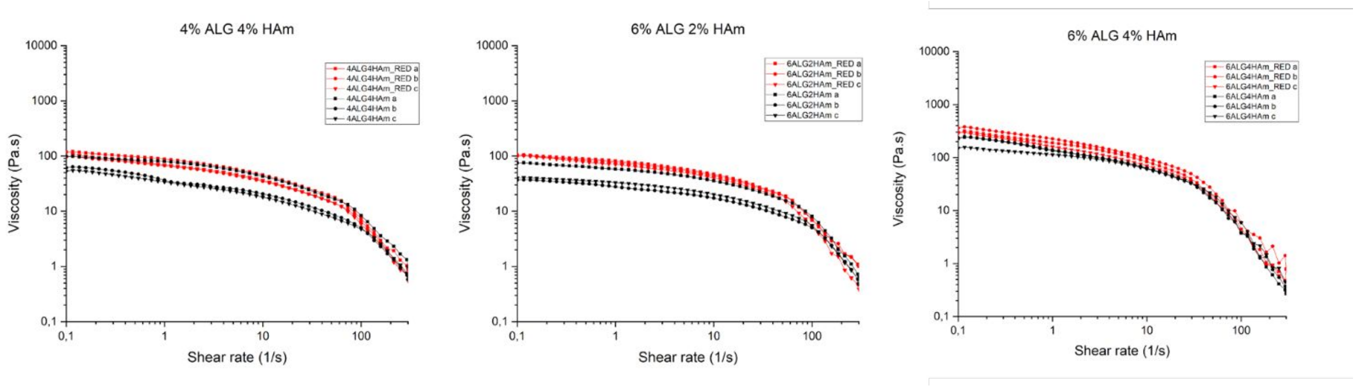

Figure. S1 Comparison of the viscosity measurements with and without red dye for conditions 4ALG4HA, 6ALG2HA and 6ALG4HA.

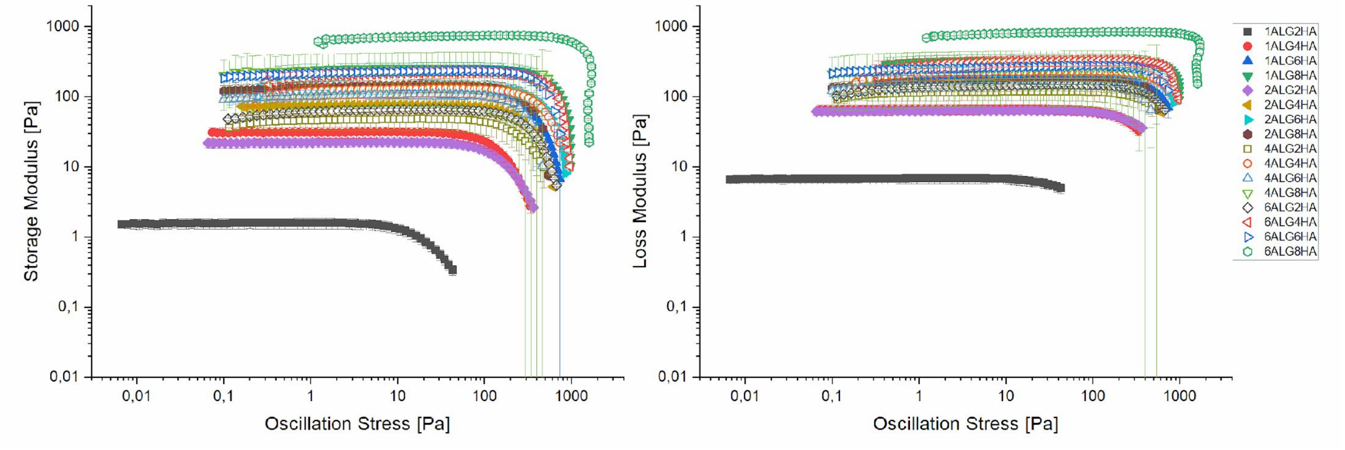

Figure. S2 Storage and loss moduli for all the tested blends. The curves are reported as the average of the three replicates for each condition with standard deviation.

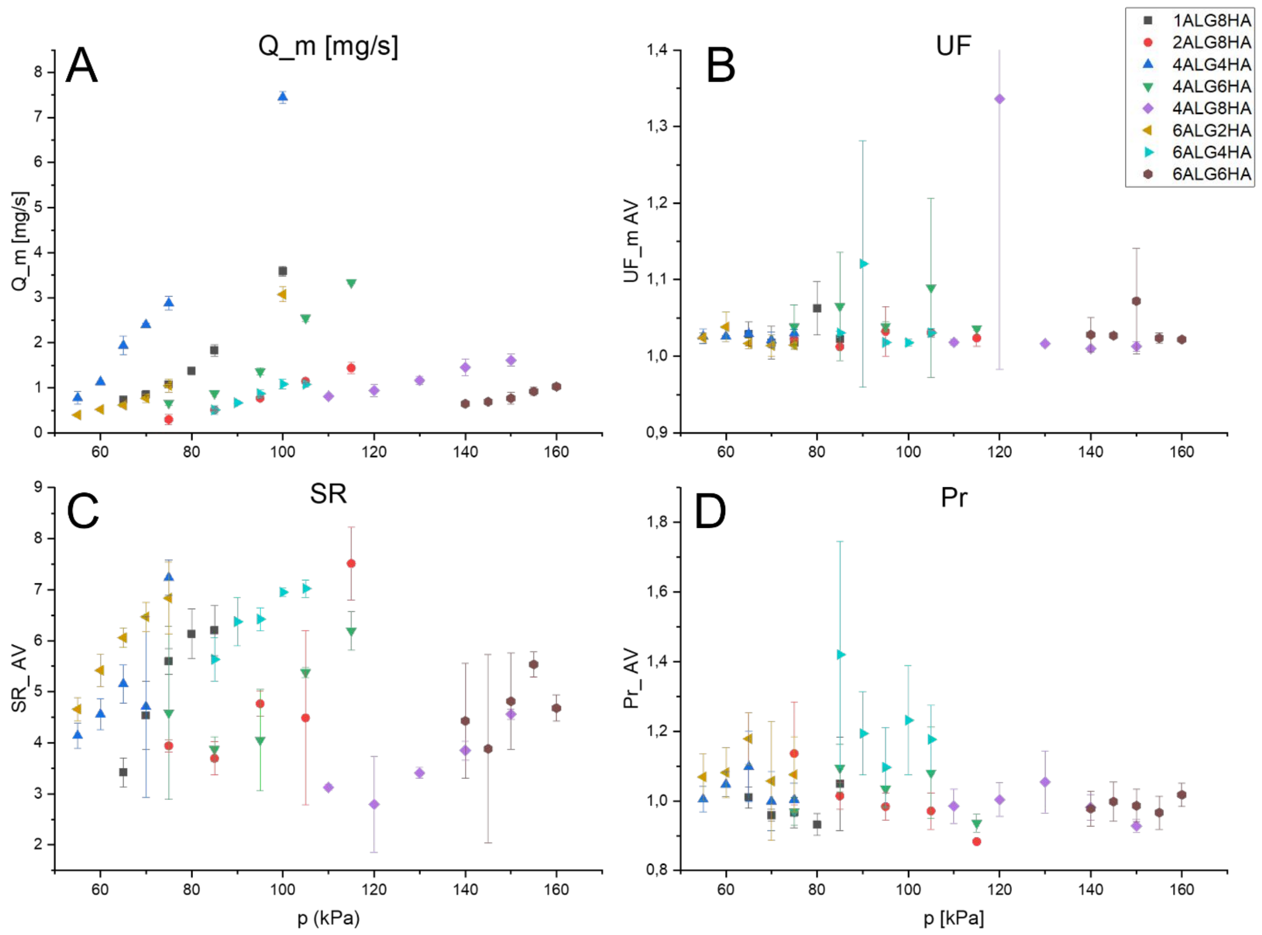

Figure. S3 **A**: Average mass flow rate of the tested concentrations as function of the extrusion pressure, **B**: Uniformity ratio of the tested concentrations as function of the extrusion pressure, **C**: Spreading ratio of the tested concentrations as function of the extrusion pressure, **D**: Printability Index of the tested concentrations as function of the extrusion pressure.

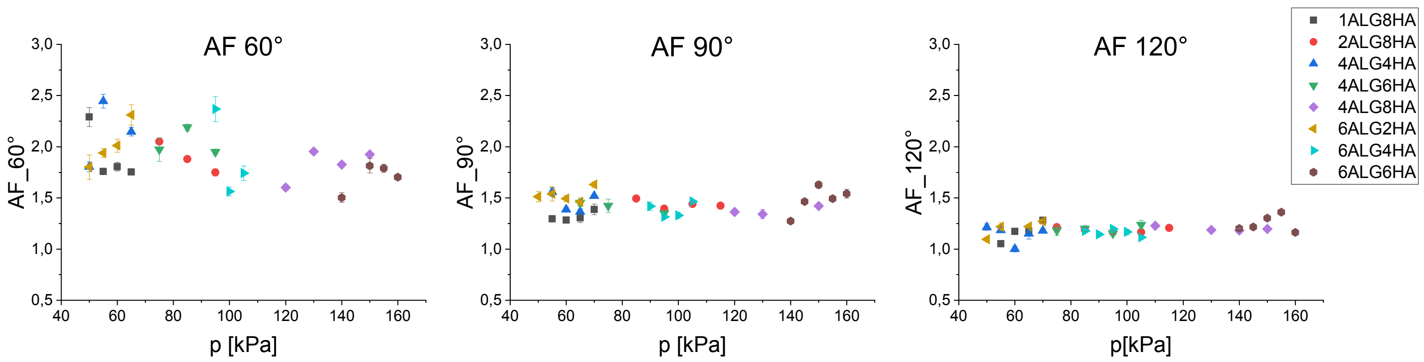

Figure. S4 Angle fidelity factor of the tested concentrations at different angles (60°, 90°, 120°) as a function of the printing pressure.

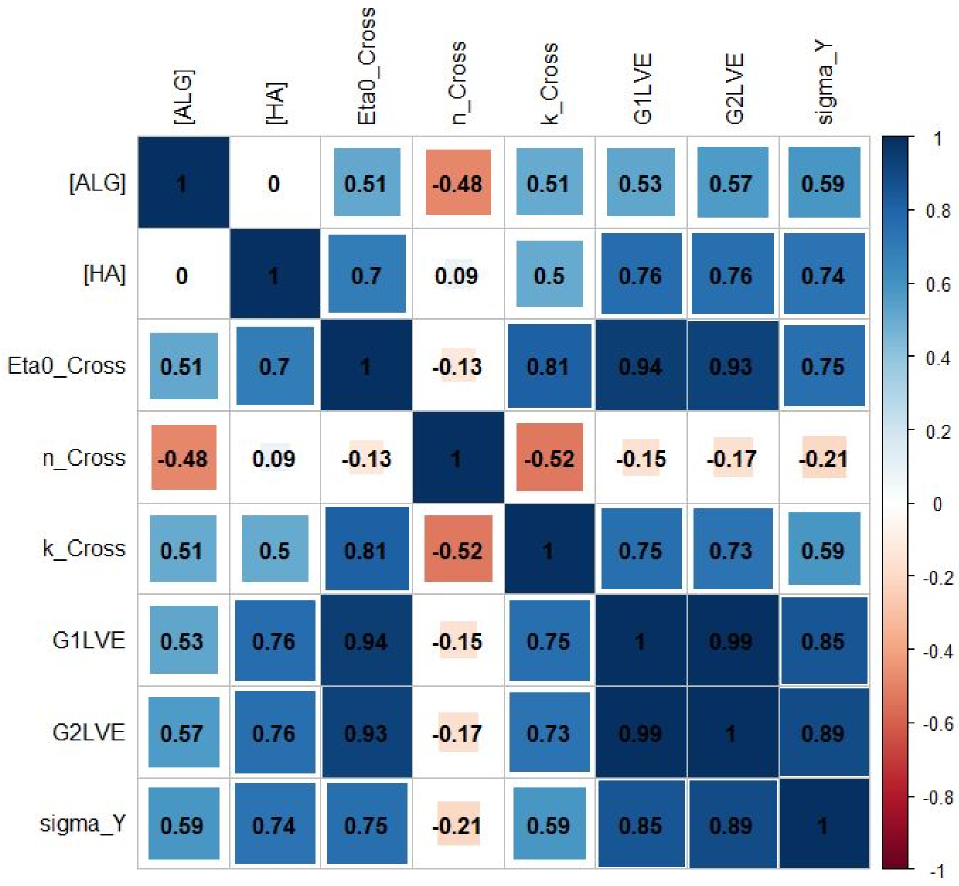

Figure. S5 Correlation Matrix of the dataset, value below 0.3 or above -0.3 indicates a non significant linear correlation between the considered couple of variables.

N	Factor 1 A:Alg mg/mL	Factor 2 B:HA mg/mL	Response 1 η_0 Pa*s	Response 2 m	Response 3 k s	Response 4 G'_LVE Pa	Response 5 G''_LVE Pa	Response 6 τ_Y Pa
1	10	80	182.904	0.766497	0.278983	198.142	256.716	611.629
2	10	80	217.307	0.767669	0.324032	309.828	403.094	534.135
3	10	80	234.41	0.80038	0.331158	275.958	359.134	565.15
4	20	80	299.799	0.81071	0.365788	348.512	453.378	522.956
5	20	80	282.775	0.746452	0.45178	366.736	465.446	490.018
6	20	80	317.925	0.653762	0.85776	351.511	449.834	528.521
7	40	40	76.477	0.464118	1.41845	58.2717	102.764	232.268
8	40	40	119.324	0.560052	0.481202	154.97	268.387	597.288
9	40	40	99.7334	0.740178	0.149962	152.103	256.375	509.356
10	40	60	303.234	0.685191	0.457944	332.033	469.823	524.477
11	40	60	298.463	0.712753	0.457501	350.419	491.155	545.868
12	40	60	245.419	0.481116	0.985485	349.103	480.041	601.69
13	40	80	429.922	0.807125	0.533299	481.729	584.872	551.896
14	40	80	627.275	0.868908	0.592477	485.604	582.931	562.351
15	40	80	720.634	0.820207	0.891118	462.978	563.69	589.076
16	60	20	42.4777	0.619607	0.123291	67.8291	151.783	344.064
17	60	20	43.6288	0.482242	0.2706	71.4151	159.942	377.903
18	60	20	79.5261	0.587807	0.156502	69.5039	157.687	319.316

19	60	40	309.773	0.579064	1.21938	282.158	409.129	579.767
20	60	40	272.174	0.619333	0.783726	220.196	342.292	527.459
21	60	40	154.963	0.705315	0.20098	215.685	305.333	550.613
22	60	60	368.777	0.876524	0.321061	409.564	548.005	728.99
23	60	60	461.591	0.874637	0.399713	465.543	618.049	812.845
24	60	60	500.218	0.832547	0.55101	583.156	731.616	740.711
25	10	40	17.3976	0.81582	0.102216	25.4874	55.5448	89.938
26	10	40	10.7996	0.752467	0.0978945	32.9381	73.0738	101.116
27	10	40	27.5373	0.994333	0.113069	36.3581	75.9214	134.286
28	10	60	85.4876	0.798231	0.162716	102.919	182.826	345.995
29	10	60	125.104	0.827367	0.196981	126.577	212.279	386.637
30	10	60	105.464	0.88239	0.204083	94.8642	159.559	274.288
31	10	20	1.07972	0.792742	0.0163589	1.24531	5.61121	9.23615
32	10	20	1.65765	0.816741	0.013645	1.7431	7.58322	11.2769
33	10	20	2.28101	0.788162	0.0213297	1.69927	7.42938	12.3149
34	20	20	9.50184	0.759762	0.0219009	19.6786	58.5346	133.526
35	20	20	11.134	0.699697	0.0262325	23.4917	67.7755	111.419
36	20	20	10.8441	0.73763	0.0335717	26.3829	73.3926	124.915
37	20	40	37.8958	0.74955	0.0779691	60.0132	129.265	182.209
38	20	40	47.0283	0.87239	0.0758585	99.3036	174.63	327.126
39	20	40	64.6457	0.952439	0.0880795	72.2622	141.713	280.449
40	20	60	75.8155	0.759901	0.140666	162.319	250.989	489.131
41	20	60	47.9806	0.674053	0.160979	152.2	241.348	471.587
42	20	60	64.008	0.847363	0.10613	113.531	186.94	419.588
43	40	20	22.294	0.728743	0.0501603	35.3013	90.6707	199.702
44	40	20	30.7534	0.832004	0.0482187	68.4632	160.751	263.201
45	40	20	27.6872	0.789437	0.046825	45.8701	116.279	200.105
46	60	80	520.664	0.616021	1.03433	799.781	888.443	841.719
47	60	80	960.43	0.627027	1.83225	799.883	892.56	737.281
48	60	80	1005.04	0.577041	2.30706	798.437	880.267	725.137

Table. S1 Data set used to fit the empirical model of the rheological properties.

Run	Factor 1 A:Alg mg/mL	Factor 2 B:HA mg/mL	Factor 3 C:P kPa	Response 1 Pr mean	Response 2 Pr StD	Response 5 Qm mean	Response 6 Qm StD	Response 7 SR mean	Response 8 SR StD
1	10	80	65	1.010	0.029	0.735	0.019	3.422	0.283
2	10	80	70	0.959	0.017	0.851	0.019	4.538	0.669
3	10	80	75	0.967	0.042	1.074	0.018	5.597	0.247
4	10	80	80	0.932	0.030	1.375	0.068	6.138	0.485
5	10	80	85	1.049	0.130	1.834	0.103	6.203	0.490
6	10	80	100			3.589	0.089		
7	20	80	75	1.136	0.143	0.293	0.095	3.943	0.124
8	20	80	85	1.014	0.037	0.507	0.011	3.697	0.329
9	20	80	95	0.984	0.038	0.767	0.034	4.767	0.248
10	20	80	105	0.971	0.051	1.144	0.027	4.490	1.711
11	20	80	115	0.883	0.002	1.438	0.102	7.511	0.716

12	40	40	55	1.005	0.035	0.778	0.112	4.142	0.245
13	40	40	60	1.048	0.034	1.134	0.006	4.559	0.305
14	40	40	65	1.098	0.099	1.797	0.171	5.156	0.373
15	40	40	70	0.999	0.081	2.403	0.028	4.706	1.769
16	40	40	75	1.004	0.045	2.881	0.125	7.238	0.350
17	40	40	100			7.447	0.105		
18	40	60	75	0.969	0.038	0.658	0.040	4.586	1.698
19	40	60	85	1.094	0.066	0.874	0.010	3.873	0.245
20	40	60	95	1.034	0.055	1.363	0.065	4.056	0.990
21	40	60	105	1.081	0.127	2.547	0.056	5.379	0.098
22	40	60	115	0.936	0.025	3.336	0.021	6.194	0.384
23	40	80	110	0.985	0.048	0.811	0.039	3.129	0.062
24	40	80	120	1.004	0.047	0.942	0.112	2.796	0.937
25	40	80	130	1.054	0.086	1.163	0.069	3.413	0.107
26	40	80	140	0.981	0.035	1.450	0.150	3.850	0.185
27	40	80	150	0.928	0.018	1.616	0.106	4.560	0.102
28	60	20	55	1.069	0.065	0.395	0.014	4.657	0.233
29	60	20	60	1.081	0.071	0.522	0.007	5.419	0.322
30	60	20	65	1.179	0.072	0.612	0.043	6.059	0.189
31	60	20	70	1.057	0.164	0.769	0.075	6.469	0.286
32	60	20	75	1.076	0.096	1.048	0.122	6.838	0.704
33	60	20	100			3.074	0.138		
34	60	40	85	1.420	0.309	0.506	0.078	5.633	0.427
35	60	40	90	1.194	0.116	0.665	0.008	6.376	0.475
36	60	40	95	1.097	0.111	0.871	0.070	6.424	0.229
37	60	40	100	1.232	0.153	1.084	0.089	6.955	0.077
38	60	40	105	1.177	0.095	1.076	0.055	7.023	0.173
39	60	60	140	0.978	0.049	0.644	0.055	4.431	1.127
40	60	60	145	0.998	0.055	0.689	0.048	3.885	1.844
41	60	60	150	0.986	0.045	0.768	0.105	4.816	0.948
42	60	60	155	0.966	0.046	0.918	0.075	5.536	0.247
43	60	60	160	1.017	0.032	1.027	0.057	4.683	0.258

Table. S2 Data set used to fit the empirical model of the 2D printing yields (Pr, Qm and SR)

Run	Factor 1 A:Alg mg/mL	Factor 2 B:HA mg/mL	Factor 3 C:P kPa	Factor 4 D:Angle °	Response 1 mean AF	Response 2 StD AF
1	10	80	50	60	2.29017	0.0936253
2	10	80	55	60	1.7596	0.0301965
3	10	80	60	60	1.80526	0.0412513
4	10	80	55	90	1.29597	0.0210961
5	10	80	65	60	1.75407	0.0158443
6	10	80	60	90	1.28243	0.029433
7	10	80	65	90	1.30412	0.0453863
8	10	80	70	90	1.38795	0.0513345

9	10	80	55	120	1.05256	0.0295853
10	10	80	60	120	1.17187	0.022162
11	10	80	65	120	1.17573	0.0194448
12	10	80	70	120	1.28156	0.0144789
13	20	80	75	60	2.04975	0.016115
14	20	80	85	60	1.87831	0.0167196
15	20	80	95	60	1.74885	0.0334194
16	20	80	85	90	1.4926	0.0162292
17	20	80	95	90	1.39505	0.0125334
18	20	80	105	90	1.44103	0.00846958
19	20	80	115	90	1.42223	0.00551604
20	20	80	75	120	1.2125	0.0185018
21	20	80	85	120	1.19111	0.0182752
22	20	80	95	120	1.16533	0.0153714
23	20	80	105	120	1.16534	0.0191255
24	20	80	115	120	1.20579	0.0158535
25	40	40	50	60	1.80422	0.0482703
26	40	40	55	60	2.44505	0.0673524
27	40	40	65	60	2.14773	0.0410755
28	40	40	55	90	1.55854	0.0404212
29	40	40	60	90	1.38725	0.0237332
30	40	40	65	90	1.36564	0.0275376
31	40	40	70	90	1.52016	0.00885943
32	40	40	50	120	1.21458	0.031865
33	40	40	55	120	1.18475	0.0251478
34	40	40	60	120	1.00068	0.028701
35	40	40	65	120	1.15143	0.053961
36	40	40	70	120	1.18045	0.020291
37	40	60	75	60	1.97211	0.115179
38	40	60	85	60	2.18925	0.0312527
39	40	60	95	60	1.94988	0.0269887
40	40	60	65	90	1.45212	0.0365994
41	40	60	75	90	1.42363	0.0644926
42	40	60	95	90	1.35048	0.0178555
43	40	60	105	120	1.23805	0.0418633
44	40	60	75	120	1.18126	0.0479477
45	40	60	85	120	1.20002	0.0268969
46	40	60	95	120	1.15453	0.0215345
47	40	80	120	60	1.60113	0.0298627
48	40	80	130	60	1.95358	0.0216747
49	40	80	140	60	1.82522	0.0133087
50	40	80	150	60	1.92546	0.0219518
51	40	80	120	90	1.36288	0.0282561
52	40	80	130	90	1.34172	0.0445454
53	40	80	150	90	1.42105	0.0145608
54	40	80	110	120	1.22687	0.0203316

55	40	80	130	120	1.18756	0.0206974
56	40	80	140	120	1.18237	0.0173148
57	40	80	150	120	1.19584	0.0204479
58	60	20	50	60	1.80075	0.120007
59	60	20	55	60	1.93806	0.0437234
60	60	20	60	60	2.0106	0.0630045
61	60	20	65	60	2.31133	0.0988328
62	60	20	50	90	1.5134	0.0504629
63	60	20	55	90	1.53985	0.0697319
64	60	20	60	90	1.49433	0.0310203
65	60	20	65	90	1.4624	0.0374672
66	60	20	70	90	1.62994	0.0274714
67	60	20	50	120	1.09488	0.0162623
68	60	20	55	120	1.21931	0.0365402
69	60	20	65	120	1.22121	0.00902439
70	60	20	70	120	1.27114	0.0290935
71	60	40	95	60	2.36855	0.123116
72	60	40	100	60	1.56292	0.0428463
73	60	40	105	60	1.7423	0.0698619
74	60	40	90	90	1.41795	0.0411236
75	60	40	95	90	1.31523	0.00945609
76	60	40	100	90	1.32876	0.0307992
77	60	40	105	90	1.46489	0.0155806
78	60	40	85	120	1.17845	0.0104487
79	60	40	90	120	1.14397	0.0103393
80	60	40	95	120	1.19772	0.0201288
81	60	40	100	120	1.16671	0.0273399
82	60	40	105	120	1.11558	0.0117484
83	60	60	140	60	1.50363	0.047354
84	60	60	150	60	1.81434	0.0715356
85	60	60	155	60	1.78987	0.0386035
86	60	60	160	60	1.70334	0.0283493
87	60	60	140	90	1.27302	0.0232889
88	60	60	145	90	1.4647	0.0146547
89	60	60	150	90	1.62833	0.0337752
90	60	60	155	90	1.49292	0.0278472
91	60	60	160	90	1.54228	0.0432433
92	60	60	140	120	1.20107	0.00993045
93	60	60	145	120	1.21688	0.0252817
94	60	60	150	120	1.30275	0.00780293
95	60	60	155	120	1.35987	0.0105086
96	60	60	160	120	1.16353	0.0154409

Table. S3 Data set used to fit the model of the angular 2D printing yields (AF)

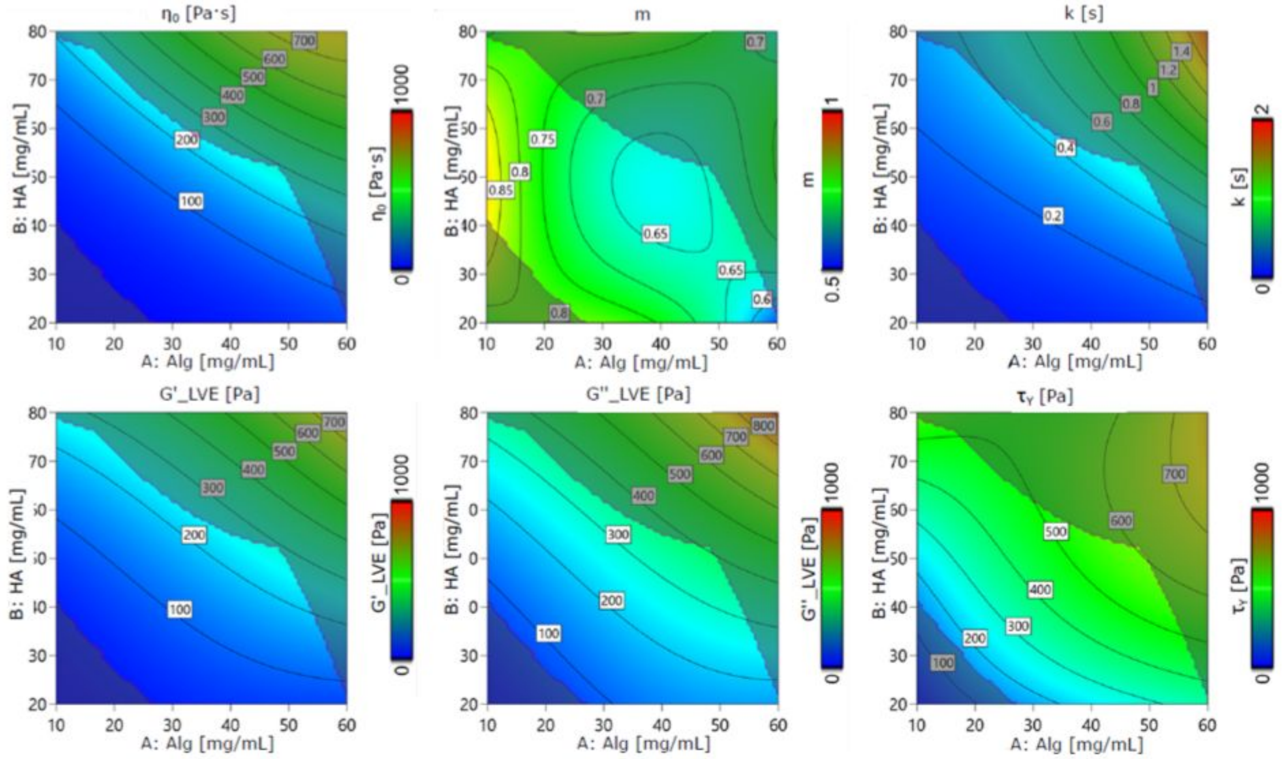

Figure. S6 Contour plot of the model predicting the rheology within the printability bands in case of 55kPa of applied pressure.

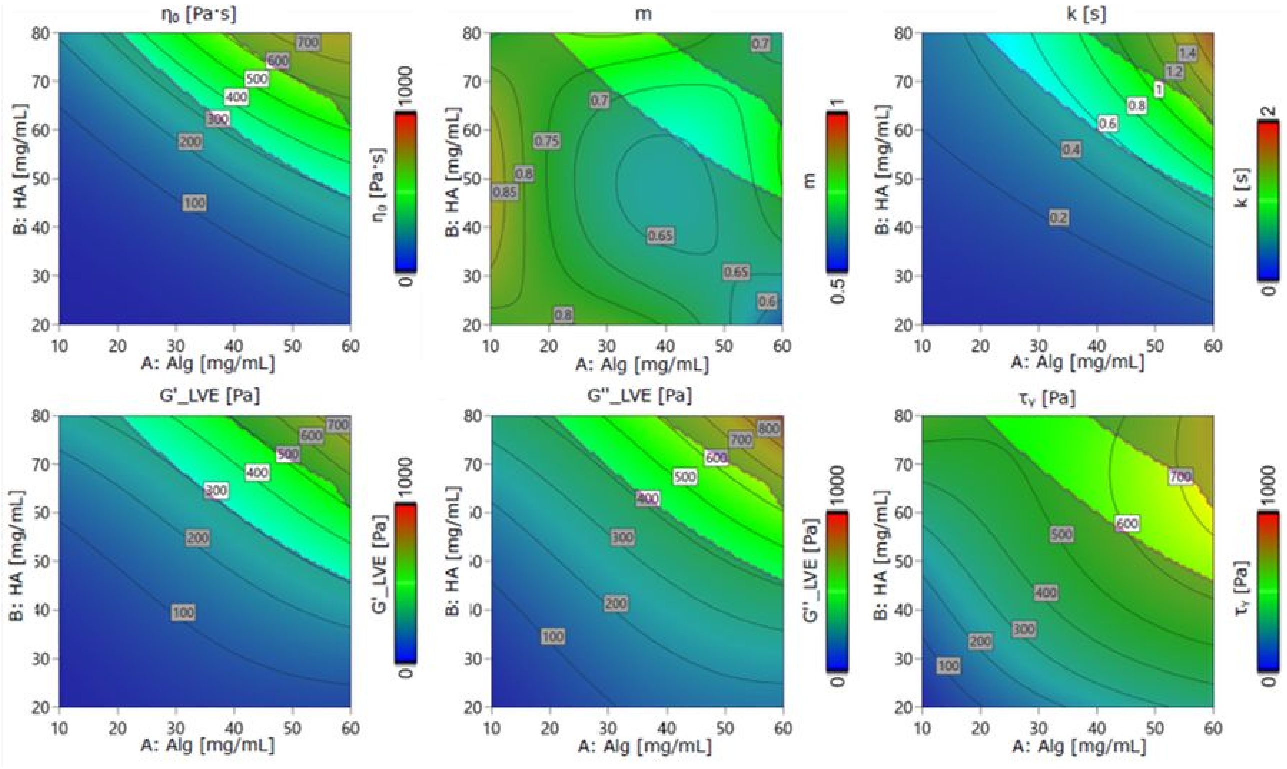

Figure. S7 Contour plot of the model predicting the rheology within the printability bands in case of 107.5kPa of applied pressure.

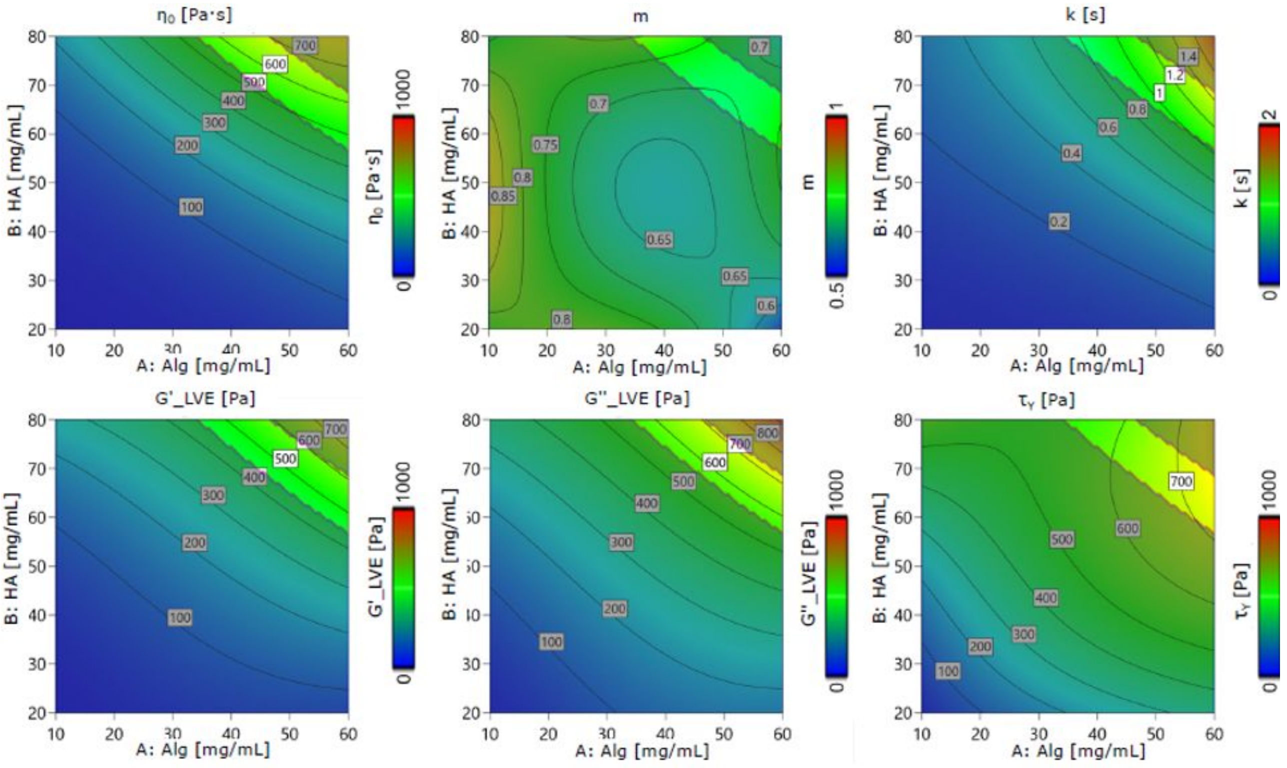

Figure. S8 Contour plot of the model predicting the rheology within the printability bands in case of 160kPa of applied pressure.

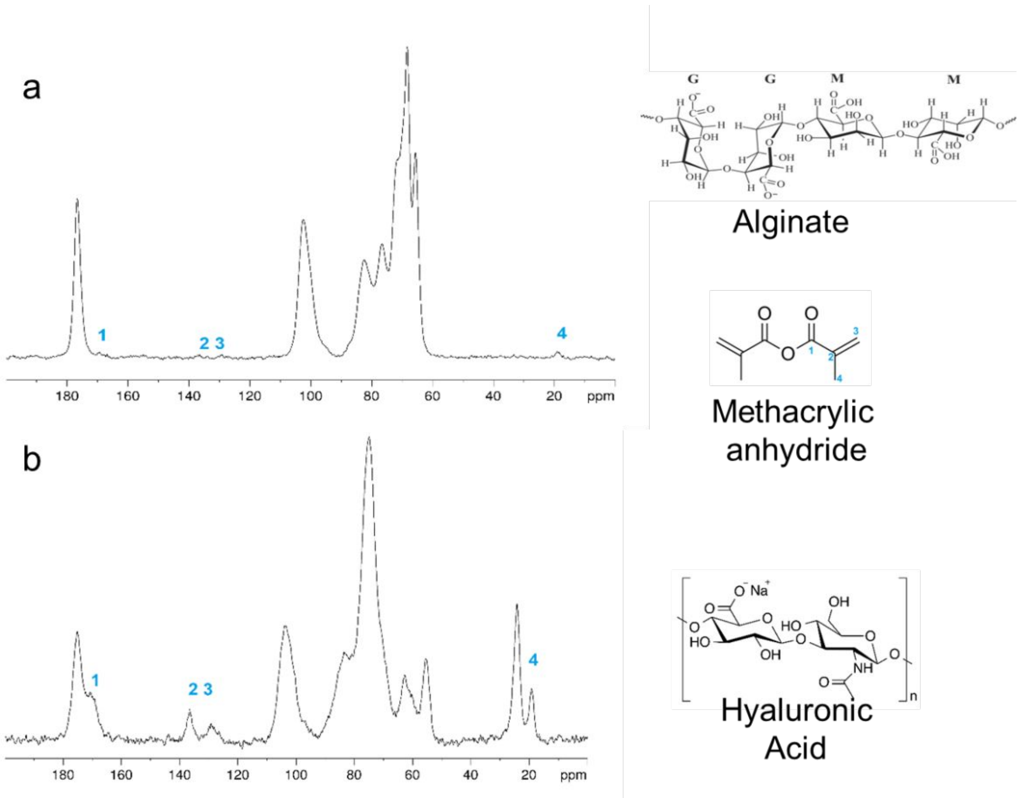

Figure. S9 ^{13}C CPMAS NMR spectra of a) AL-MA and b) HA-MA. The numbers refer to the anhydride in the scheme to the right. Solid state NMR analyses were carried out with a Bruker

Avance 400WB spectrometer. NMR spectra were acquired with cp pulse sequences under the following conditions: 13C frequency: 100.48 MHz, contact time 2000 µs, decoupling length 6.3 µs, recycle delay: 5s, 2k scans. Samples were packed in 4 mm zirconia rotors, which were spun at 9 kHz under air flow. Adamantane was used as an external secondary reference.

**δ (ppm)**	**176 / Area**	**169 / Area**
AL-MA	100	1.1
HA-MA	100	29.6

Table. S4 The integrals of the two carbonyl belonging to the saccharides at about 176 ppm and the methacrylate at 169 ppm are taken into account to quantify the methacrylation degree. It should be mentioned that the ^{13}C CPMAS experimental parameters were selected to obtain quantitative results. In the case of AL-MA the amount of methacrylate units can be also assessed by comparison between the integral of the 5 ring carbons and the area of the anhydride methyl at 19 ppm. Similarly, for HA-MA it is possible to better compare the integrals of the two methyls at 24 and 19 ppm due to hyaluronic acid and anhydride, respectively. Both evaluations confirm the results presented.

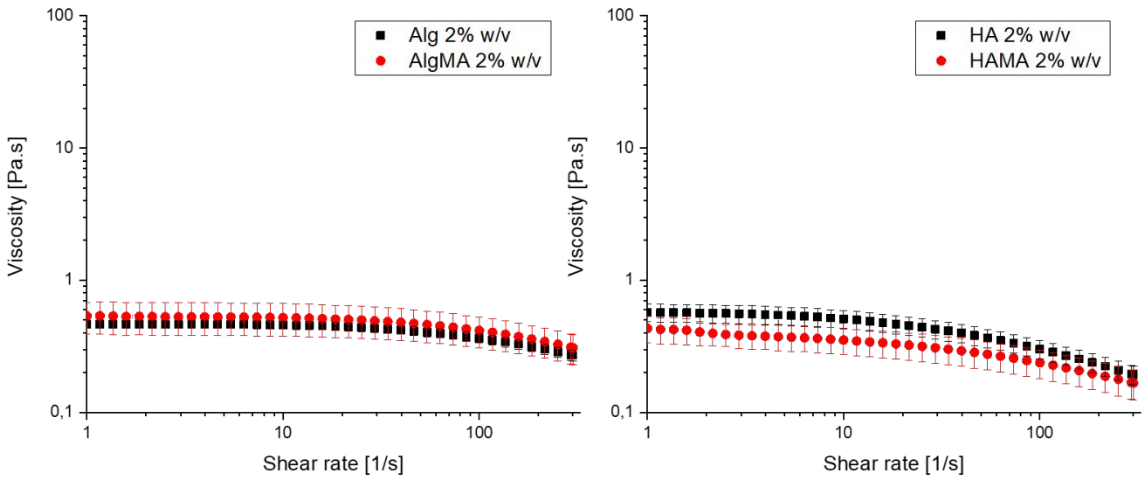

Figure. S10 Viscosity measurement comparison of alginate and hyaluronic acid solutions 2% w/v and their methacrylated counterparts.

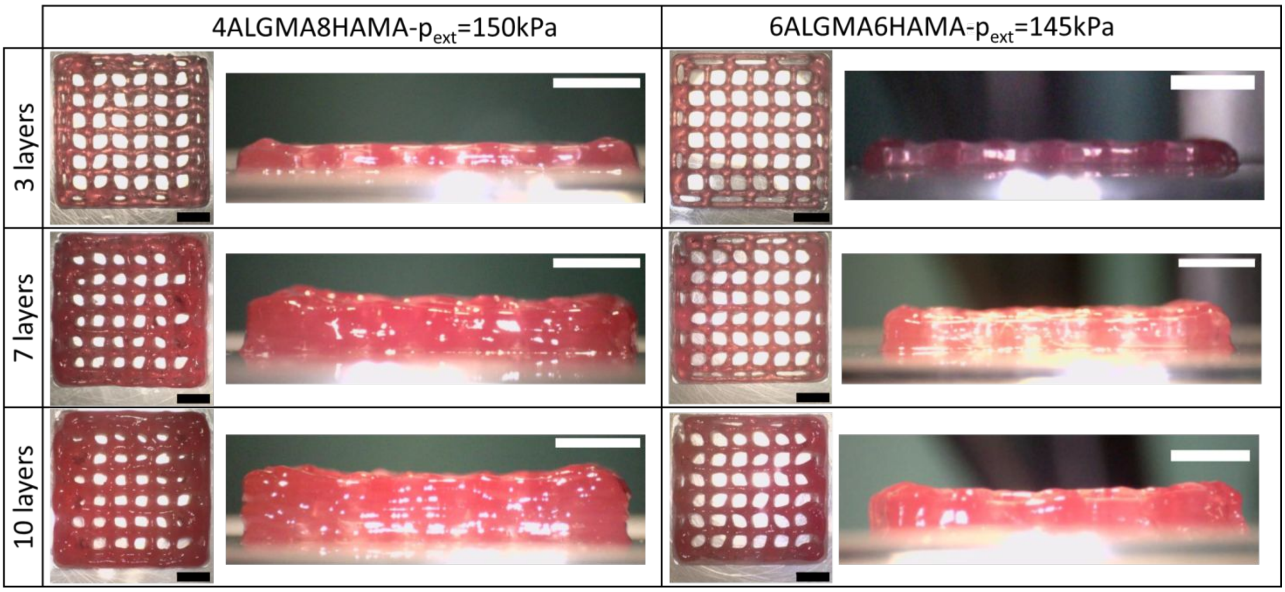

Figure. S11 Pictures of some multilayered methacrylated blend grids with two different compositions and printing pressures, printed with 3, 7 and 10 layers. All the reference bars are 5mm.

**Source type**	BioX printer left module
**Wavelenght**	365nm
**Distance from source to construct**	3cm
**Intensity**	11.6 mW/cm2
**Exposure time for each layer**	3s

Table. S5 Parameters for the photocrosslinking of the methacrylated multilayered constructs
